# Drosophila Casein Kinase I Alpha Regulates Homolog Pairing and Genome Organization by Modulating Condensin II Subunit Cap-H2 Levels

**DOI:** 10.1371/journal.pgen.1005014

**Published:** 2015-02-27

**Authors:** Huy Q. Nguyen, Jonathan Nye, Daniel W. Buster, Joseph E. Klebba, Gregory C. Rogers, Giovanni Bosco

**Affiliations:** 1 Geisel School of Medicine at Dartmouth, Hanover, New Hampshire, United States of America; 2 Department of Cellular and Molecular Medicine, University of Arizona Cancer Center, University of Arizona, Tucson, Arizona, United States of America; Stowers Institute for Medical Research, UNITED STATES

## Abstract

The spatial organization of chromosomes within interphase nuclei is important for gene expression and epigenetic inheritance. Although the extent of physical interaction between chromosomes and their degree of compaction varies during development and between different cell-types, it is unclear how regulation of chromosome interactions and compaction relate to spatial organization of genomes. Drosophila is an excellent model system for studying chromosomal interactions including homolog pairing. Recent work has shown that condensin II governs both interphase chromosome compaction and homolog pairing and condensin II activity is controlled by the turnover of its regulatory subunit Cap-H2. Specifically, Cap-H2 is a target of the SCF^Slimb^ E3 ubiquitin-ligase which down-regulates Cap-H2 in order to maintain homologous chromosome pairing, chromosome length and proper nuclear organization. Here, we identify Casein Kinase I alpha (CK1α) as an additional negative-regulator of Cap-H2. CK1α-depletion stabilizes Cap-H2 protein and results in an accumulation of Cap-H2 on chromosomes. Similar to Slimb mutation, CK1α depletion in cultured cells, larval salivary gland, and nurse cells results in several condensin II-dependent phenotypes including dispersal of centromeres, interphase chromosome compaction, and chromosome unpairing. Moreover, CK1α loss-of-function mutations dominantly suppress condensin II mutant phenotypes *in vivo*. Thus, CK1α facilitates Cap-H2 destruction and modulates nuclear organization by attenuating chromatin localized Cap-H2 protein.

## Author Summary

The Cap-H2 condensin II subunit is required for interphase condensin II activity. Previous work has shown that low levels of Cap-H2 protein in interphase is achieved by SCF^Slimb^ mediated protein turnover and limits chromatin-bound protein levels. Here we show that Casein Kinase I alpha (CK1α) is also a negative regulator of interphase condensin II activity by promoting Cap-H2 destruction and limiting chromatin-bound Cap-H2 levels. Loss of CK1α function leads to aberrant chromosome structures that are suppressed by mutation or depletion of Cap-H2 or other condensin subunits. These observations suggest that normally, interphase condensin II levels must be kept low in order to maintain proper interphase chromosome organization, and this low activity is maintained by targeted destruction of Cap-H2.

## Introduction

Interphase genome organization in eukaryotic cells is non-random [1,2,3]. Indeed, organization of the genome is crucial because it influences nuclear shape and processes such as DNA repair and replication, as well as gene expression [4, 5, 6]. While chromosomes are highly organized within the nucleus, they must also remain extremely dynamic. Chromosome dynamics facilitate events that occur not only during cell division, but also during interphase, when cells respond to developmental and environmental cues that require changes in gene expression. Interphase events include trans-interactions such as homolog pairing, chromosome remodeling and compaction, and DNA looping. Although numerous studies using Fluorescent In-Situ Hybridization (FISH), live cell imaging, and chromosome conformation capture techniques have revealed the three-dimensional (3D) organization of genomes, much remains to be discovered regarding the factors that govern the overall conformation of interphase chromosomes. An equally important task is to identify the molecular mechanisms that regulate and maintain specific 3D genome organizational states.

Condensin complexes are highly conserved from bacteria to humans [7,8,9] and have been identified as key drivers of genome organization [10]. Eukaryotes have two condensin complexes, condensin I and II, which share the core SMC2 and SMC4 (Structural Maintenance of Chromosomes) subunits but differ in their non-SMC Chromosome Associated Protein (CAP) subunits. Condensins have long been known to play vital roles in shaping mitotic chromosomes. While condensin I promotes lateral chromosome compaction, condensin II promotes axial compaction; both of which are necessary for faithful mitotic condensation and chromosome segregation [11]. Condensins also display different localization patterns: condensin I only associates with mitotic chromosomes, whereas condensin II is present in the nucleus, where it is bound to chromatin throughout the cell cycle [12,13,14,15]

In Drosophila cells, condensin II performs a variety of functions during interphase, including chromosome compaction, unpairing of homologous chromosomes, and driving the formation and maintenance of chromosome territories. The condensin II subunit, Cap-H2, has been shown to be the rate limiting subunit, as overexpression of Cap-H2 results in the increase of condensin II chromosome activity [16,17] [18] [19,20] [21]. Furthermore, all of these Drosophila Cap-H2 functions were shown to either require or be dependent on one or more other condensin subunits, such as SMC4, SMC2 and/or Cap-D3. This strongly suggests that these Cap-H2 functions are likely to be performed in the context of an active condensin II complex. Drosophila condensin II also plays an essential function in anaphase-I of male meiosis, where it is thought to be required for resolving entanglements between homologs as well as heterologs [17]. Strikingly, loss or depletion of condensin II function leads to lengthening of interphase chromosomes, suggesting that chromosome axial compaction must be actively maintained even after exit from mitosis [21,22]. Additionally, Drosophila Cap-D3 regulates the expression of immunity genes while repressing somatic cell transposon activation, although it is unclear if these effects are linked to condensin mediated compaction activity [23,24].

In vertebrates, condensin II is required for axial compaction of chromosomes and sister chromatin resolution in S-phase [15,25]. Loss of the Cap-D3 condensin II subunit also leads to loss of compaction in S-phase [26]. That interphase chromosome compaction levels are important for gene expression has been recently highlighted in mammalian cells. Condensin II has been found to regulate the STAT5 transcription factor by highly condensing interphase chromatin during T-cell differentiation; failure to repress condensin II activity prevents STAT5 access to its binding sites, resulting in cells that are unable to differentiate normally [27]. Similarly, the bromodomain protein Brd4, recruits condensin II to chromatin and local condensation serves to attenuate signaling from the damaged DNA sites [28]. Thus physical compaction of interphase chromatin can be an effective mechanism for limiting transcription factor binding as well as modulating important signaling.

While it is evident that the role of condensin II in interphase genome organization, DNA repair, and gene expression is important in both vertebrate and Drosophila cells, how condensin II executes these functions is not well understood. In fact, it is unknown whether processes such as anti-pairing, gene regulation, and transposon repression are secondary effects to its potential primary role in shaping chromatin architecture. Moreover, the molecular mechanism regulating interphase condensin II remains unclear. Therefore, elucidating these regulatory mechanisms will lead to important insights into how interphase genome organization is modulated.

Previously, it was demonstrated that overexpression of the condensin II regulatory subunit, Cap-H2, was sufficient to drive both interphase compaction and the unpairing of homologous chromosomes in vivo and in cultured cells [16,20,21,22]. In contrast, mutations that inactivate other condensin II genes, by decreasing their dosage or depleting their expression by RNAi, suppress all Cap-H2 overexpression phenotypes. These observations strongly suggest that 1) Cap-H2-induced homolog anti-pairing and compaction is reliant on a functional condensin II complex, and 2) that Cap-H2 is the rate-limiting component in the activation of the catalytic SMC2/4 subunits, which are present during interphase and are able to bind chromatin. Thus, Cap-H2 availability controls condensin II activity, and consequently, Cap-H2 protein levels and chromatin localization represent key steps for SMC2/4 regulation in interphase. This idea is consistent with the observation that Cap-H2 loading onto chromatin is partially dependent on the chromodomain protein Mrg15 [21]. Moreover, Cap-H2 protein levels are controlled by the SCF^Slimb^ ubiquitin-ligase, maintaining low levels of Cap-H2 in vivo and in cultured Drosophila cells [20]. Interestingly, Slimb recognizes its target proteins through a phosphodegron motif [29], suggesting that one or more kinases must phosphorylate Cap-H2 before Slimb can target it for destruction. A Slimb-binding site consensus sequence (DSGXXS) exists in the extreme C-terminus of Cap-H2 and deletion of this region renders Cap-H2 non-degradable [20]. As expected for a Slimb substrate, Cap-H2 protein mobility on SDS-PAGE was sensitive to phosphatase treatment, suggesting that Cap-H2 is phosphorylated [20].

Given that Cap-H2 protein levels may be regulated by its phosphorylation state, we set out to identify kinases that target Cap-H2 for Slimb recognition and that lead to its degradation. We show that in Drosophila cultured S2 cells, Casein Kinase I alpha (CK1α) depletion results in the hypercondensation of interphase chromatin in a condensin II-dependent manner. We also found that CK1α and condensin II genetically interact in vivo, and that CK1α depletion leads to Cap-H2 protein enrichment on polytene and cultured cell chromosomes. Similar to Slimb depletion [20], CK1α depletion also results in stabilization of Cap-H2 protein in cultured cells. Our findings further elucidate the mechanism by which Cap-H2, and thus condensin II, is regulated and contribute significantly to our understanding of how interphase genome organization, homolog pairing, and chromosome compaction is modulated.

## Results

### Casein Kinase I alpha is required for interphase chromatin reorganization

**Fig 1:**
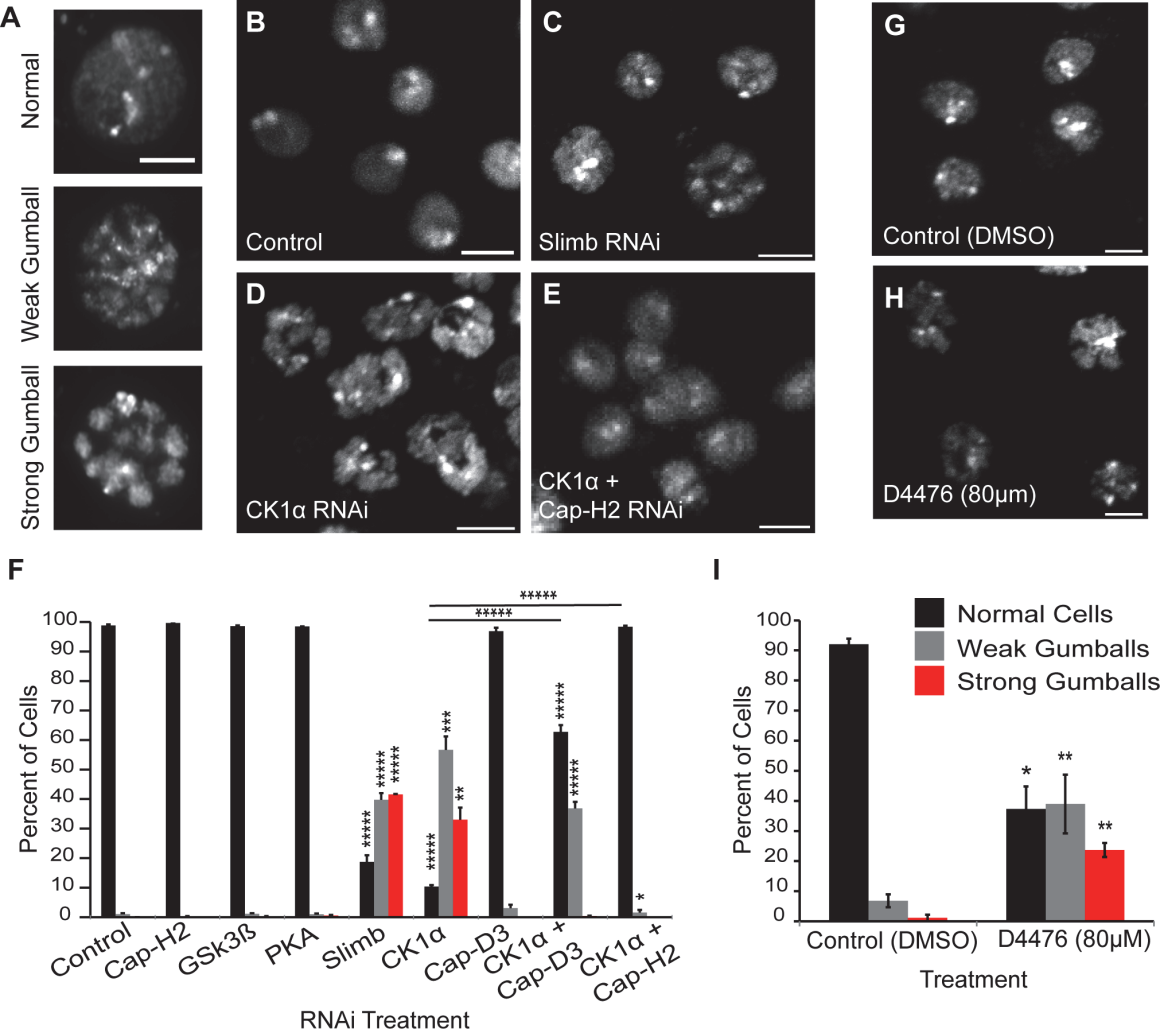
CK1α inactivation alters interphase chromosome morphology. (A) Representative micrographs of *Drosophila* cultured S2 cells displaying different chromatin-gumball phenotype classifications. Scale, 2.5μm. (B-E) Micrographs of 6 day RNAi-treated Kc cells stained with DAPI to visualize DNA. Depletion of Slimb (C) or CK1α (D) but not control (B) promotes the chromatin-gumball phenotype, while double RNAi with CK1α and Cap-H2 (E) suppresses this phenotype. Scale, 5μm. (F) Frequency histogram of the nuclear phenotypes in S2 cells after 6-day depletion of the indicated proteins via RNAi; (n = 2200–4200 cells per treatment). (G-H) Micrographs of S2R+ cells stained with DAPI to visualize DNA. Treatment of cells with the CK1 inhibitor D4476 (80μM) (H) for 8 hours promotes chromatin-gumball phenotype not observed with control DMSO treatment (G). Scale, 5μm. (I) Frequency histogram of the nuclear phenotypes in S2R+ cells after treatment with control (DMSO) or CK1 inhibition (D4476); (n = 110–170 cells per treatment). p-value = * < 0.05, ** < 0.01, *** = < 0.005, **** = < 0.001, ***** = < 0.0001 (calculated by using students’ t-test in Microscoft excel). Horizontal lines on histograms represent comparisons between percentage of normal cells of two treatments at either end of the line. Error bars in all figures indicate SEM. (A-E,G-H) Maximum projection image of multiple z-slices.

Previously, we discovered that the Cap-H2 subunit of condensin II is a SCF^Slimb^ ubiquitination-target in Drosophila cells [20]. In a whole genome RNAi screen, Slimb was also identified as a homolog pairing-promoting factor, and it was shown to affect pairing in a Cap-H2 dependent manner[18]. In cultured S2 and Kc cells, depletion of SCF^Slimb^ components Slimb, Cul-1 and SkpA prevents Cap-H2 degradation and leads to condensin II hyperactivation during interphase and the remodeling of each chromosome into a compact globular structure (Fig. 1A-C). Based on their overall appearance, we refer to these hypercondensed chromosomes as “chromatin-gumballs” (Fig. 1A). Overexpression of a GFP tagged wild type Cap-H2 also induces this phenotype [20]. Since phosphorylation of the Slimb-binding domain within its substrates is required for Slimb binding [29], we reasoned that depletion of a kinase involved in this pathway would also stabilize Cap-H2 and phenocopy the effect on chromatin remodeling observed after Slimb depletion.

We first analyzed S2 cells that were depleted of either: Glycogen Synthase Kinase 3 Beta (GSK3β), Protein Kinase A (PKA), or Casein Kinase I alpha (CK1α). We chose these three kinases because they typically phosphorylate Slimb targets to initiate their degradation [30] [31,32] [33,34]. Strikingly, depletion of only CK1α resulted in a dramatic increase in the formation of chromatin-gumballs in interphase cells that was comparable to Slimb depletion (Fig. 1F). CK1α depletion was verified indirectly by assessing Armadillo (Drosophila β-catenin) protein levels, as the use of CK1α antibodies to Drosophila CK1α [35] and commercially available anti-human CK1α were both unsuccessful. CK1α and Slimb function to negatively regulate Armadillo, therefore we probed Armadillo protein levels to confirm that CK1α and Slimb were efficiently being depleted in our RNAi treatments, as previously shown [32,34]. In addition to the S2 cells, identical gumball phenotypes were observed in CK1α-depleted Kc cells, as previously observed in Slimb depleted cells (Fig. 1B–D). CK1α depletion in S2 cells induced chromatin-gumball formation (weak and strong) to levels significantly higher (p < 7.5x10^−8^) than control-treated cells (CK1α RNAi: 89 ±4%; control RNAi: 1 ±0.17%; sum percentage of weak and strong gumballs) (Fig. 1F). To rule out the possibility that the observed gumball phenotypes in the CK1α depleted cells were a result of apoptosis, we assessed cell viability in our Kc cell RNAi treatments. CK1α did not increase cell death over that of control treated cells (control RNAi: 18 ±2.4%; CK1α RNAi: 16.7 ±3.9%) (S1A and B Fig.). In addition to RNAi, we also inactivated CK1α in cultured S2R+ cells with the cell permeable CK1 chemical inhibitor D4476 (Fig. 1G-H) [36,37]. We observed that D4476 significantly increased (p < 0.01) the proportion of chromatin-gumball formation (weak and strong) compared to DMSO-treated control cells (DMSO: 7.9 ±1.5%, D4476: 62 ±6%; sum percentage of weak and strong gumballs) (Fig. 1I). Thus, similar to SCF^Slimb^, CK1α depletion by RNAi or pharmacological inhibition with the D4476 leads to global chromatin remodeling.

We next tested whether the CK1α RNAi-induced gumball phenotype is dependent on condensin II activity. To test this, we co-depleted both CK1α and the condensin II non-SMC subunits Cap-D3 or Cap-H2 in both Kc and S2 cells (Fig. 1E-F). Co-depletion of CK1α with either subunits resulted in a significant suppression (CK1α + Cap-D3 RNAi: p < 0.0005, CK1α + Cap-H2 RNAi: p < 3.9x10^−6^; comparison between percentage of normal cells) of the chromatin-gumball phenotype. Interestingly, CK1α co-depletion with Cap-D3 did not suppress the gumball phenotype as well as co-depletion with Cap-H2 (CK1α + Cap-D3 RNAi: 62 ±2.3% normal nuclei, CK1α + Cap-H2 RNAi: 98 ±0.35% normal nuclei) (Fig. 1F). These observations suggest that in the absence of CK1α, condensin II-mediates hyper-compaction of interphase chromosomes.

### CK1α prevents dispersal of centromeres

**Fig 2:**
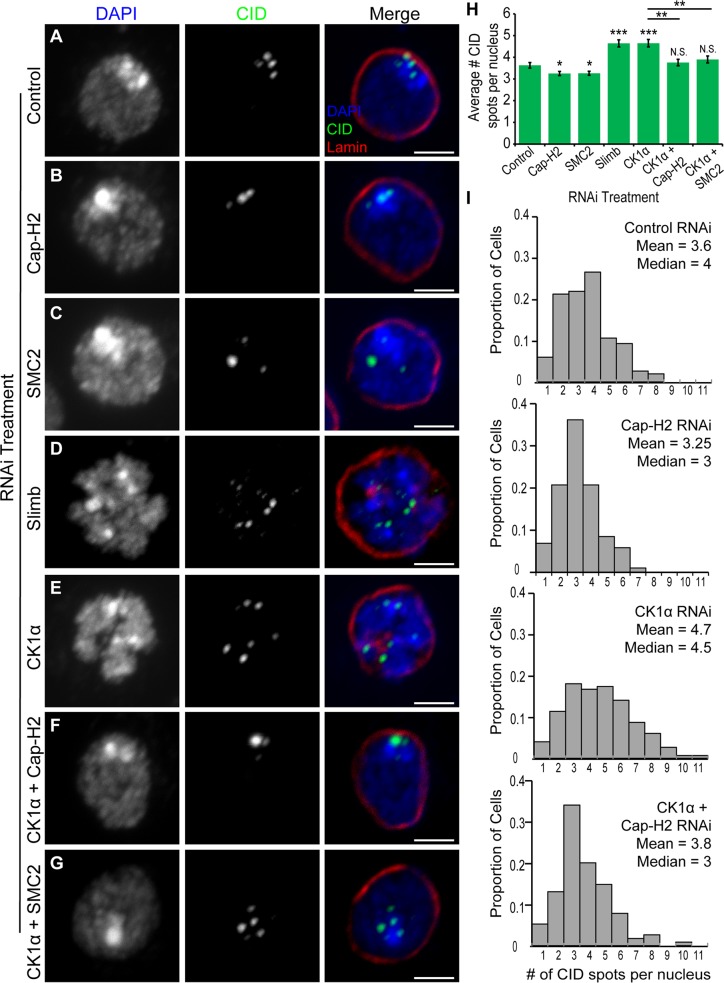
RNAi of CK1α leads to dispersal of centromeres in Kc cells. (A-G) RNAi treated Kc cells immunostained for centromeric protein (CID, green), Lamin Dm0 (red), and counterstained for DNA (DAPI, blue). CK1α RNAi (E) induces abnormal CID dispersal similar to Slimb RNAi (D), which is suppressed by double RNAi of CK1α + Cap-H2 (F) or CK1α + SMC2 (G). Scale, 2.5μm. (H) Histogram showing average number of CID spots per nucleus after RNAi depletion of the indicated protein. Slimb and CK1α depletion results in a significant increase in average number of CID spots per nucleus; (n = 115–180 cells per treatment). (I) Histograms showing the distribution of CID spots per nucleus. CK1α depletion results in an increased proportion of cells with higher than normal CID spots; (n = 115–150 cells per treatment). N.S. = No significance. p-value = * < 0.05, ** < 0.005, *** < 0.0001 (calculated by using students’ t-test in Microscoft excel). Statistical comparisons are between RNAi treatments and control, unless denoted by horizontal line between bars. Error bars in all figures indicate SEM. (A-G) Maximum projection image of multiple z-slices.

One function of condensin II in cultured cells and in vivo is to drive dispersal of centromeres, as visualized by immunostaining of Centromeric Identifier (CID) protein or by FISH to pericentric heterochromatin[20,22]. It has been proposed that centromere dispersal is a direct consequence of chromosome compaction [18,22]. The results from our RNAi experiments suggest that CK1α may be functioning to repress Cap-H2, as co-depletion of CK1α with Cap-H2 strongly suppresses the gumball phenotype (Fig. 1E-F). Stabilization of Cap-H2 protein levels, if functional, is expected to drive dispersal of centromeric regions and result in a greater number of CID foci in each nucleus. To test this hypothesis, cultured Drosophila cells (Kc and S2) were depleted of CK1α *via* RNAi treatment and immunostained using an antibody specific to CID. The number of CID spots per nucleus was counted, with an increase in CID spots per nucleus indicating an increase in centromere dispersal. CID spots in control treated cells appear clustered, whereas CK1α depletion results in CID signal dispersal and a significant increase (p < 3x10^−6^) in the number of CID spots (for Kc cells, CK1α RNAi: 4.7 ±0.17 and Control RNAi: 3.6 ±0.13 spots per nucleus) (Fig. 2A,E,H-I). Furthermore, this increase in CID dispersal was suppressed when either condensin subunits SMC2 or Cap-H2 were co-depleted with CK1α (CK1α + SMC2 RNAi: 3.9 ±0.16 and CK1α + Cap-H2 RNAi: 3.76 ±0.15 spots per nucleus) (Fig. 2F-I). In addition, co-depletion of the Condensin I specific subunit Barren (Drosophila Cap-H) with CK1α did not suppress the increase in CID dispersal (CK1α + Barren RNAi: 4.8 ±0.18 spots per nucleus) (S2C Fig.). Similar to Slimb, CK1α acts as an inhibitor of condensin II mediated centromere dispersal (Fig. 2D-E,H). This was also observed in S2 cells (S2A Fig. and B). To exclude the possibility that the increases in CID dispersal may be explained by an increase in cell ploidy, DNA content in RNAi treated cells was analyzed by flow cytometry. Flow cytometry on S2 cells demonstrates that CK1α depletion slightly increases the proportion of cells in G1 (CK1α RNAi: 51.5% and Control RNAi: 42.4%) (S3C Fig.), therefore, the increase in number of CID foci in CK1α RNAi cells is not due to increases in centromere numbers resulting from polyploidy. These results indicate that CK1α is normally acting to inhibit condensin II dependent centromere dispersal.

### CK1α antagonizes condensin II mediated chromosome axial compaction

**Fig 3:**
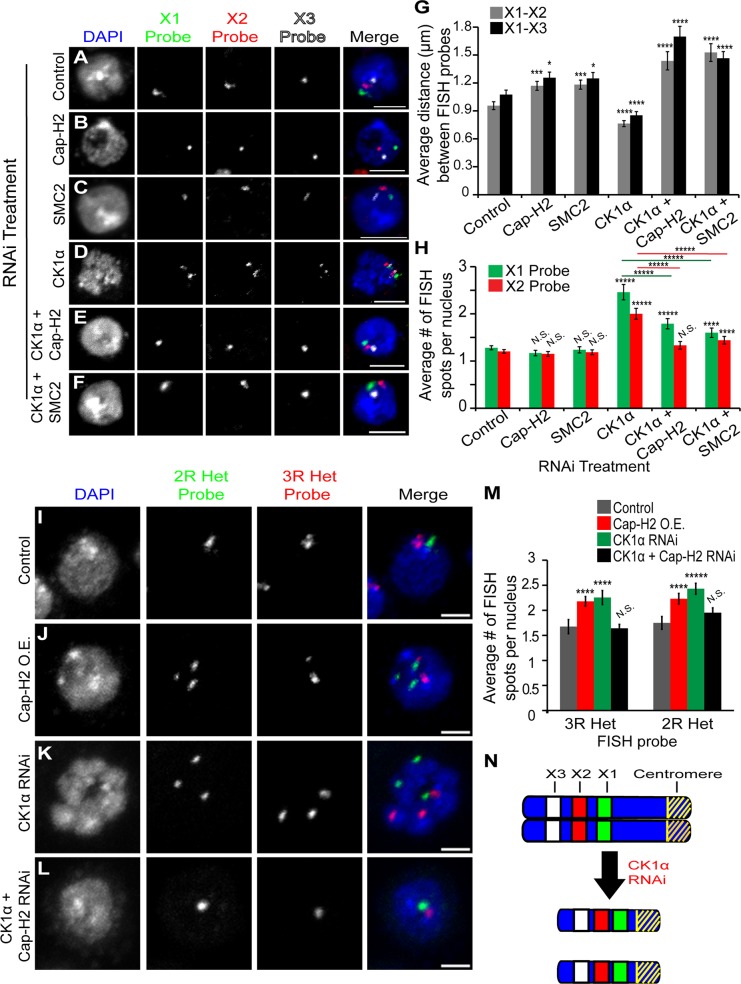
CK1α depletion increases chromosome compaction and unpairing activity in Kc cells. (A-F) Micrographs of RNAi treated Kc cells stained for DAPI (blue) and three different euchromatic FISH probes specific for the X chromosome (green, red, and white). Single FISH spot for each probe signifies that the homologs are paired and multiple FISH spots indicate the unpairing of chromosomes. Scale, 5μm. (G) Histogram showing the average 3D distance between pairwise FISH spots in microns in RNAi treated Kc cells (n = 45–100 cells per treatment). CK1α RNAi significantly reduces the distance between FISH spots, and this reduction is suppressed by double RNAi of CK1α + Cap-H2 or CK1α + SMC2. (H) Histogram showing the average number of FISH spots per nucleus in RNAi depleted Kc cells (n = 50–110 cells per treatment). CK1α RNAi significantly increases the number of FISH spots, and this increase is suppressed by double RNAi of CK1α + Cap-H2 or CK1α + SMC2. (I-L) Micrographs of RNAi treated Kc cells stained with FISH probes specific to heterochromatin on Chromosome 2R (green) and 3R (red) and counterstained for DNA (DAPI, blue). Cap-H2 overexpression (J) and CK1α depletion (K) induces unpairing of heterochromatin, which is suppressed by double RNAi of CK1α + Cap-H2 (L). Scale, 2.5μm. (M) Histogram showing average number of heterochromatin FISH spots per nucleus after RNAi depletion of the indicated protein. CK1α depletion results in a significant increase in number of FISH spots; (n = 40–78 cells per treatment). (N) Diagram of X chromosomes. Blue represents the DNA, yellow diagonal stripes represents the centromere, and green (X1), red (X2), and white (X3) rectangles represent FISH probes used for compaction and unpairing experiments. Depletion of CK1α via RNAi results in compaction and unpairing of chromosomes. N.S. = No significance. p-value = * < 0.05, ** < 0.01, *** < 0.005, **** < 0.001, ***** < 0.0001 (calculated by using students’ t-test in Microscoft excel). Error bars in all figures indicate SEM. (A-F,I-L) Maximum projection image of multiple z-slices.

In addition to promoting the dispersal of centromeric regions, Cap-H2 has been shown to be important for maintenance of interphase chromosome axial length [21,22]. If CK1α is a negative regulator of Cap-H2, then CK1α depletion should lead to an increase in chromosome compaction and a decrease in axial length. To measure chromosome compaction, we performed 3D DNA FISH in RNAi treated cultured cells using three probes specific to euchromatic loci on the X chromosome (Fig. 3). FISH probes were designed approximately 2Mb apart. We found that CK1α depletion resulted in a significant decrease in pairwise 3D distances between FISH probes compared to control treated cells (X1-X2 = p < 0.0004, X1-X3 = p < 0.001) (Fig. 3A,D,G). In control treated cells, the distance between X1 and X2 probes was 0.96 ±0.04μm and the distance between X1 and X3 probes was 1.08 ±0.05μm. CK1α depletion caused these distances to decrease about 20% to 0.76 ±0.05μm between X1 and X2 probes and 0.85 ±0.04μm between X1 and X3 probes. This increase in chromosome compaction resulting from depletion of CK1α suggests that CK1α normally antagonizes chromosome compaction. Interestingly, CK1α co-depletion with condensin subunits SMC2 or Cap-H2 increased the axial length of chromosomes, relative to control treated cells (CK1α + SMC2 RNAi: X1-X2 = 1.5 ±0.1μm and X1-X3 = 1.4 ±0.07μm, CK1α + Cap-H2 RNAi: X1-X2: 1.4 ±0.1μm and X1-X3 = 1.7 ±0.1μm) (Fig. 3E-G). We noted that the axial chromosome length seen with co-depletion of CK1α with SMC2 or Cap-H2 was significantly increased compared to depletion of Cap-H2 or SMC2 alone (p < 0.05 for X-chromosome probes X1-X2 and X1-X3, Fig. 3G). It is unclear why co-depletion of CK1α and codensin II subunits would lead to the observed axial lengthening that is greater than in control cells.

We considered the possibility that CK1α depletion led to a mitotic arrest phenotype where chromosome compaction status could be due to mitotic condensation. Control cells and CK1α depleted cells were stained with anti-lamin and the mitotic marker anti-phospho-histone H3 (phospho-H3) to assess whether the RNAi treated cells were undergoing mitosis. We found that the number of phospho-H3 positive cells in CK1α depleted cells was not significantly increased compared to control cells. In fact, CK1α depleted cells showed a significant decrease in phospho-H3 positive cells (CK1α RNAi = 0.71 ±0.12%, Control = 2.15 ±0.11%; p < 3.36x10^−7^) (S3A Fig. and B). Furthermore, nuclear envelope staining by anti-lamin and DNA visualization by DAPI staining both indicated that the vast majority (>95%) of cells examined were in interphase. Lastly, flow cytometry measuring DNA content of RNAi treated S2 cells also demonstrated that there was only moderate differences in cell cycle profile of CK1α depleted and CK1α, Cap-H2 co-depleted cells compared to control RNAi treated cells (S3C Fig.). Thus, these data demonstrating that CK1α functions to inhibit chromosome compaction in cultured cells provide strong evidence that CK1α limits interphase condensin II activity, and that these compaction differences cannot be explained by dramatic shifts in cell cycle distribution.

### CK1α promotes homologous chromosome pairing

Cap-H2 and other condensin II subunits have been shown to function as factors that antagonize homologous chromosome pairing during interphase [16,18,19]. In contrast, Slimb has been identified as a pairing-promoting factor that negatively regulates the Cap-H2 anti-pairing activity [18,20]. Therefore, we predicted that CK1α also is a pairing promoting factor not identified in previous chromosome pairing screens. To test this, we performed FISH in RNAi treated Kc cells using FISH probes designed to label two different euchromatic loci on the X chromosome. Homolog pairing can be assessed by counting the number of fluorescent spots per nucleus for each of the two probes. A single spot for each FISH probe signifies chromosomes are paired, whereas two spots signify unpairing of homologs. Normally, homologs are paired in most Drosophila cells, and in Kc cells at levels of 85% or greater [18,38]. CK1α depletion results in an increase in unpairing of chromosomes compared to control cells, evident by the significant increase (p < 1.3x10^−8^) in number of FISH spots counted per nucleus (control RNAi: X1 = 1.3 ±0.04 and X2 = 1.2 ±0.04, CK1α RNAi: X1 = 2.5 ±0.16 and X2 = 2 ±0.11 spots per nucleus) (Fig. 3A,D,H and S2D Fig.). To test whether this increase in unpairing was condensin dependent, cells were treated with RNAi to both CK1α and Cap-H2 or CK1α and SMC2 (Fig. 3E-F). Depletion of either Cap-H2 or SMC2 suppressed the increased unpairing caused by CK1α RNAi (CK1α + Cap-H2 RNAi: X1 = 1.8 ±0.1 and X2 = 1.3 ±0.08, CK1α + SMC2 RNAi: X1 = 1.6 ±0.08 and X2 = 1.4 ±0.08 spots per nucleus) (Fig. 3H). We also depleted the condensin I specific subunit, Barren (Cap-H), along with CK1α and found that co-depletion did not significantly suppress the increased unpairing seen with CK1α RNAi (CK1α + Barren RNAi: X1 = 2.3 ±0.1 and X2 = 2 ±0.1 spots per nucleus) (S2D Fig. and E). This indicates that adding a double-stranded RNA targeting CK1α and a second gene does not lead to suppression of anti-pairing activity simply by diluting the CK1α RNAi-depletion. Instead, this strongly suggests that the pairing promoting function of CK1α is likely due to its ability to repress condensin II anti-pairing activity.

In addition to the X1 and X2 probes specific to euchromatic loci, FISH probes specific to heterochromatic sequences on the right arms of chromosomes 2 and 3 (2R and 3R) were used to assess the pairing status of heterochromatin. Similar to the observations at the euchromatic loci, FISH using heterochromatin probes exhibited the same behavior, showing that CK1α promotes pairing at euchromatin and heterochromatin. Depletion of CK1α significantly increased (p < 0.005) the number of spots per nucleus for each of the two heterochromatic FISH probes used, compared to control treated cells (CK1α RNAi: 3R = 2.25 ±0.14 and 2R = 2.43 ±0.11, control RNAi: 3R = 1.68 ±0.14 and 2R = 1.75 ±0.13 spots per nucleus) (Fig. 3I,K,M and S2F Fig.). This increase in heterochromatin unpairing was similar to that observed when an inducible GFP tagged version of full-length Cap-H2 was expressed in cells (Cap-H2 O.E.: 3R = 2.18 ±0.1, 2R = 2.23 ±0.11 spots per nucleus) (Fig. 3J,M and S2F Fig.). The increase in unpairing of both euchromatic and heterochromatic loci after CK1α depletion is consistent with what is observed when Cap-H2 activity is increased and provide further evidence that CK1α functions as a pairing promoting factor by antagonizing condensin II anti-pairing function.

### CK1α maintains polytene chromosome pairing in larvae

**Fig 4:**
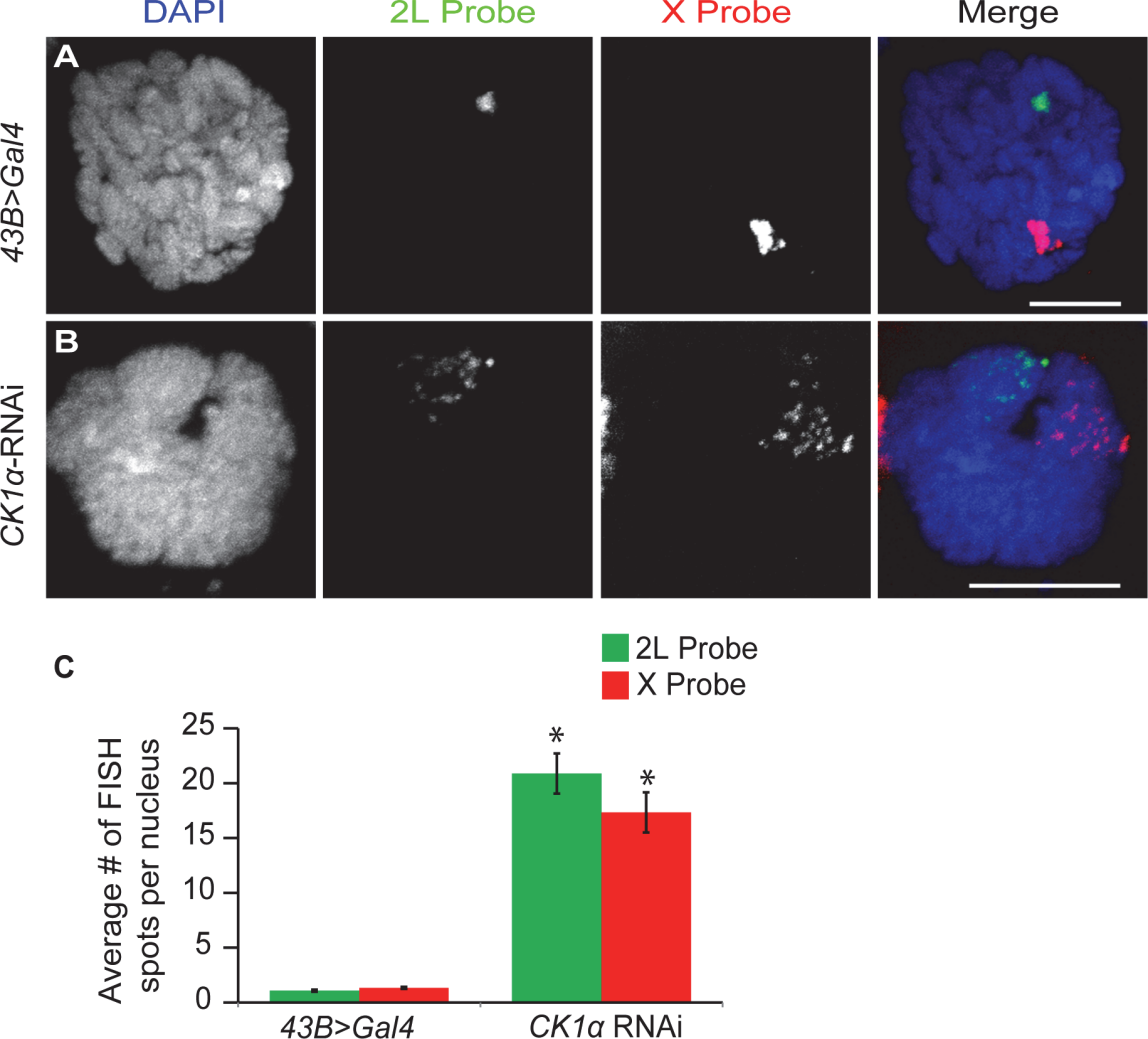
CK1α is required for polytene pairing in *Drosophila* salivary glands. (A-B) Salivary gland nuclei from control larvae (*43B>Gal4*; Gal4 under a salivary gland specific driver) (A) and larvae expressing hairpin RNAi to CK1α (*CK1α* ^*RNAi*^) driven by 43B>Gal4 (B) were hybridized with FISH probes specific to a region of Chromosome 2L (green) and Chromosome X (red) and counterstained with DAPI (blue). Chromosomes are highly paired in control (fewer FISH foci) (A) nuclei whereas expression of a TRiP hairpin RNAi targeting *CK1α* (B) induces unpairing of the chromosomes (multiple FISH foci). (C) Histogram showing average number of FISH spots per nucleus after CK1α RNAi is expressed in the salivary glands. CK1α depletion results in an increase in number of FISH spots for both 2L and X probes; (n = 24–38 nuclei per genotype). *p-value < 7.2x10^−7^ (calculated by using students’ t-test in excel). Error bars indicate SEM. (A-B) DAPI channel image is a single z-slice from the nucleus and FISH channel images are from maximum projection image of multiple z-slices. Scale, 10μm.

Based on the increased level of chromosome unpairing observed in CK1α depleted cultured cells, we wanted to test whether CK1α is repressing condensin II mediated chromosome organization in vivo. In order to do this, we turned to the salivary glands of Drosophila third instar larvae. Salivary gland nuclei contain polytene chromosomes that are highly paired. We have previously shown that overexpression of Cap-H2 is sufficient for driving unpairing of homologs and sister chromatids that are normally paired into polytenes chromosomes[16], and Slimb-RNAi depletion resulted in the disruption of polytene chromosomes[20]. If CK1α is negatively regulating Cap-H2 in vivo, then RNAi depletion of CK1α is expected to also drive polytene disruption, as has been shown for Slimb RNAi in the salivary gland. To test this, we performed FISH on whole mount salivary glands from third instar larvae, using the number of FISH spots as a direct measure of chromatid and homolog pairing (increased FISH spots indicates increased unpairing). We used FISH probes specific to euchromatic regions on chromosome X and the left arm of chromosome 2 to assay pairing at two loci. We found that salivary glands depleted of CK1α displayed a significant increase (p < 7.2x10^−7^) in the number of FISH spots compared to the controls (*43B>Gal4* driver without CK1α-RNAi) with both FISH probes (Control: X = 1.3 ±0.09 and 2L = 1.1 ±0.04, CK1α RNAi: X = 17.3 ±1.83 and 2L = 20.9 ±2.94 spots per nucleus) (Fig. 4). The increase in the number of FISH spots indicates that polytene chromosomes are unpaired in the CK1α RNAi expressing larvae. These data demonstrate that RNAi depletion of CK1α in vivo leads to increased unpairing of polytene chromosome, consistent with CK1α being a negative regulator of condensin II anti-pairing activity. Note that these FISH probes cannot distinguish between unpairing of chromatids and unpairing of homologs, and therefore we infer that disruption of paired homologous sequences includes chromatin fibers from both sisters and homologs.

These data suggest that CK1α and Slimb may function together to antagonize condensin II activity. Therefore, it is possible that CK1α, Slimb double mutants phenocopy Cap-H2 overexpression phenotypes. Because both CK1α and Slimb genes are essential for viability making homozygous mutants of either gene is not possible. However, given that Slimb heterozygotes can rescue condensin II partial loss of function phenotypes[20], we speculated that CK1α/+; Slimb/+ double heterozygous mutants may be sufficient to produce Cap-H2 gain-of-function phenotypes. To investigate whether CK1α and Slimb genetically interact to produce Cap-H2 gain-of-function phenotypes we created flies that were heterozygous for both CK1α and Slimb and measured chromosome pairing levels. For these experiments, we used two different Slimb heterozygous mutant alleles: *Slimb* ^*UU11*^/+ (null mutant) or *Slimb* ^*3A1*^/+ (loss of function), in a CK1α heterozygous mutant (*CK1α* ^*8B12*^/+, null mutant) background. We did not observe a significant increase in number of FISH spots (increased unpairing) in salivary glands of larvae heterozygous for both CK1α and Slimb, when compared to wild-type and single heterozygous mutants (for 2L probe, *Oregon-R*: 1.21 ±0.08, *CK1α* ^*8B12*^/+: 1.15 ±0.06, *Slimb* ^*UU11*^/+: 1.2 ±0.07, *Slimb* ^*3A1*^/+: 1.07 ±0.05, *CK1α* ^*8B12*^/+; *Slimb* ^*UU11*^/+: 1.19 ±0.16, *CK1α* ^*8B12*^/+; *Slimb* ^*3A1*^/+: 1.09 ±0.05 spots per salivary gland nucleus, S4 Fig.). Additionally, an increase in FISH spots was not observed in CK1α or Slimb single heterozygous mutant larval polytenes, when compared to a wild-type control (*Oregon-R*). Thus, reducing either Slimb or CK1α dosage by half or reducing dosage of both by half is not sufficient to produce condensin II gain-of-function phenotypes in salivary glands. This may be because in salivary glands, having one normal copy of CK1α and/or Slimb is sufficient to maintain normal chromosome pairing status.

### Cap-H2 and CK1α genetically interact to regulate chromosome pairing in polyploid nurse cells

**Fig 5:**
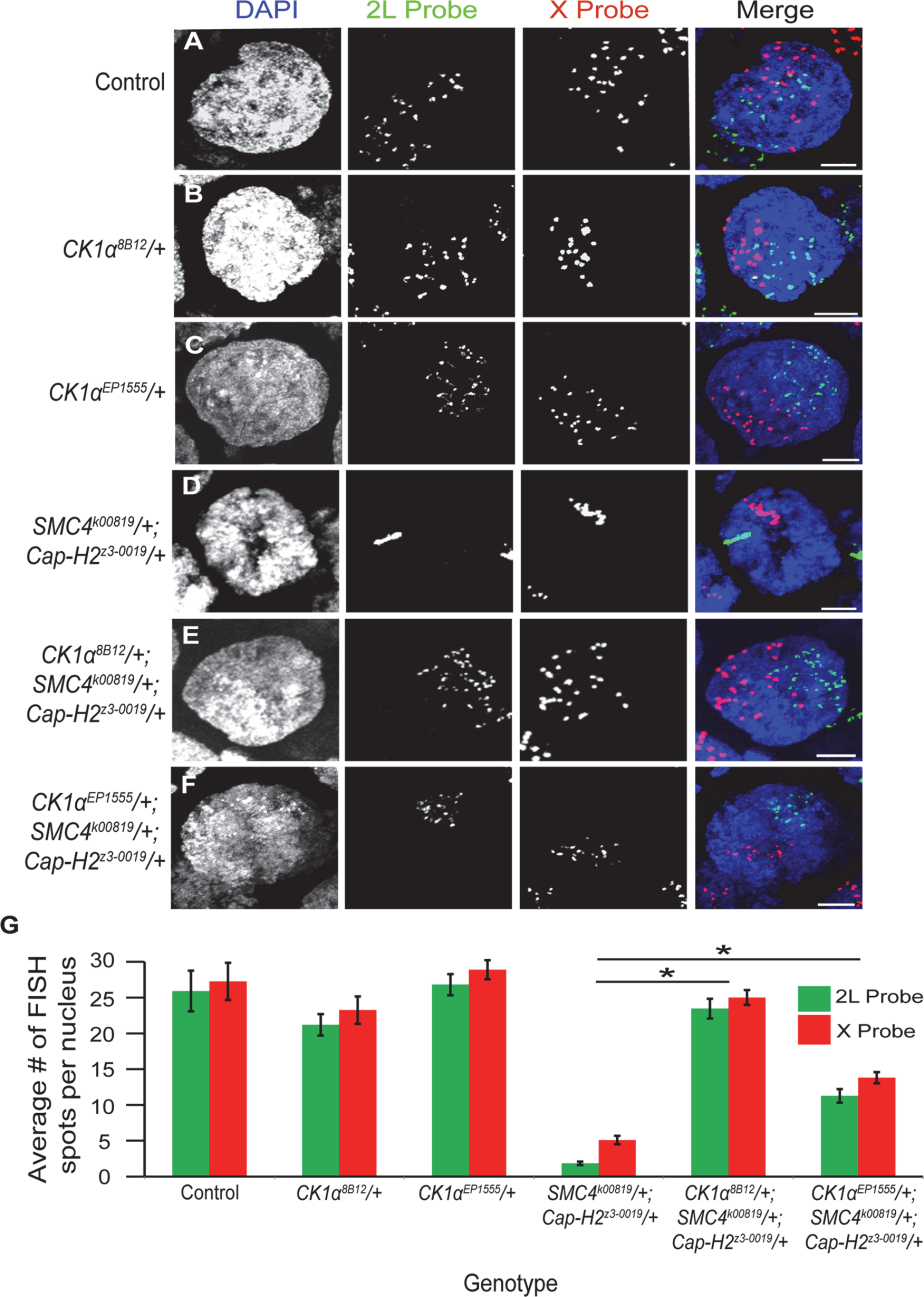
CK1α mutations suppress condensin II loss of function unpairing phenotype in polyploid nurse cells. (A-F) Micrographs of stage 10 nurse cell nuclei from control (triple balancer) (A), *CK1α^8B12^*/+ heterozygote mutant (B), *CK1α^EP1555^*/+ heterozygote mutant (C), condensin II double heterozygous mutant *SMC4^k00819^*/+; *Cap-H2^z3–0019^*/+ (D), *CK1α^8B12^*/+ heterozygote in condensin II double heterozygous background (*CK1α^8B12^*/+*; SMC4* *^k00819^*/+; *Cap-H2^z3–0019^*/+) (E), and *CK1α^EP1555^*/+ heterozygote in condensin II double heterozygous background (*CK1α^EP1555^*/+*; SMC4^k00819^*/+; *Cap-H2^z3–0019^*/+) (F) were stained with FISH probes specific to Chromosome 2L (green) and Chromosome X (red) and counterstained with DAPI (DNA, blue). Condensin II loss of function mutants (*SMC4^k00819^*/+; *Cap-H2^z3–0019^*/+) (D) show a defect in chromosome unpairing (fewer FISH spots), which is strongly suppressed when a *CK1α* ^8B12^/+ heterozygous mutation (E) is introduced into this background (dispersal of FISH spots). *CK1α* ^EP1555^/+ shows similar but weaker suppression when introduced into condensin II double heterozygous background (F). (G) Histogram showing the average number of FISH spots for each probe in stage 10 nurse cells (n = 15–26 nurse cells per genotype). Error bars indicate SEM. *p-value < 2.2x10^−10^ (calculated by using students’ t-test in MS Excel). (A-F) Maximum projection image of multiple z-slices. Scale, 10μm.

To test whether CK1α and Cap-H2 genetically interact in other tissues, we examined polytene chromosome pairing in the Drosophila ovary. Ovarian nurse cells are highly polyploid, and these polyploid chromosomes switch from a highly paired state (polytene) to an unpaired state during stage 5/6 of development [39]. We have previously shown that condensin II and, more specifically, Cap-H2, is required for this developmental switch in pairing state [16]. Flies homozygous for Cap-H2 mutations fail to unpair their chromosomes at this stage 5-to-6 transition. Combining a Cap-H2 heterozygous mutation (*Cap-H2* ^*z3–0019*^ */+*) with an SMC4 heterozygous mutation (*SMC4* ^*k00819*^ */+*) also results in an intermediate chromosome unpairing defect. We tested whether the introduction of a mutation in CK1α into this double heterozygous (*SMC4* ^*k00819*^/+; *Cap-H2* ^*z3–0019*^/+) background would modify the condensin II nurse cell unpairing defect. The *CK1α* ^*8B12*^ allele is a missense mutation in the *CK1α* gene that transforms a glycine into an aspartic acid within the kinase domain and is thought to be a null allele [40]. We hypothesized that loss of about half the normal gene dose of normal kinase activity in the CK1α heterozygous mutant may lead to sufficient stabilization of endogenous Cap-H2 protein. Stabilization of Cap-H2 is predicted to suppress the polytene unpairing defect observed in the *SMC4* ^*k00819*^/+; *Cap-H2* ^*z3–0019*^/+ double heterozygous mutants. To test this, we used FISH probes to X chromosome and 2^nd^ chromosome sequences on whole-mount ovaries and examined stage 10 nurse cells, as previously described [20,21]. We chose to examine stage 10 nurse cells specifically because any defects in unpairing should be evident at this later developmental stage. Control flies wild-type for all three genes displayed 25.9 ±2.83 spots for the 2L probe and 27.3 ±2.59 spots for the X probe per nurse cell nucleus (Fig. 5A,G). Nurse cells from flies harboring a heterozygous *CK1α* ^*8B12*^ mutation alone did not significantly increase the number of FISH spots (2L = 21.2 ±1.5 and X = 23.3 ±1.9 spots per nurse cell nucleus) (Fig. 5B,G), likely due to the chromosomes already being unpaired to their maximal degree at this developmental stage. In flies with double heterozygous mutations in condensin II (*SMC4* ^*k00819*^/+; *Cap-H2* ^*z3–0019*^/+), the stage 10 nurse cell nuclei displayed 1.9 ±0.2 and 5.1 ±0.6 spots per nurse cell nucleus for the 2L and X probe, respectively (Fig. 5D,G). This significant decrease (p < 4x10^−8^) in number of FISH spots represents a failure of these chromosomes to unpair, consistent with our previous findings [16]. However, introducing a *CK1α* ^*8B12*^/+ heterozygous mutant allele into the double heterozygous condensin II mutant background (*SMC4* ^*k00819*^/+; *Cap-H2* ^*z3–0019*^/+) increased the number of FISH spots back to wild type levels (2L = 23.5 ±1.4 and X = 25 ±1.0 spots per nurse cell nucleus; p-value compared to condensin II double mutant: p < 1.4x10^−12^; p-value compared to control: p = 0.44) (Fig. 5E,G). This triple heterozygous mutant (*CK1α* ^*8B12*^ */+; SMC4* ^*k00819*^/+; *Cap-H2* ^*z3–0019*^/+) was able to completely suppress the unpairing deficiency seen in the condensin II mutants, suggesting that reducing CK1α dosage to half is sufficient to suppress the stage 10 nurse cell condensin II-dependent polytene unpairing defects. This genetic interaction is consistent with CK1α functioning as a negative regulator of condensin II activity.

In addition to the *CK1α^8B12^* allele, we also tested two other *CK1α* heterozygous mutants. The *CK1α^EP1555^* allele is a *P*-transposable element insertion into the promoter of *CK1α* [41]. The *CK1α^G0492^* allele [42] is a CK1α mutation derived from a P-element lacW insertion into the *CK1α* gene. When crossed into the condensin II double heterozygous mutant background (*SMC4^k00819^*/+; *Cap-H2^z3–0019^*/+), both the *CK1α^EP1555^* and the *CK1α^G0492^* mutants significantly suppressed the nurse cell chromosome unpairing defect (*CK1α^EP1555^* */+; SMC4^k00819^*/+; *Cap-H2^z3–0019^*/+: 2L = 11.3 ±1.0 and X = 13.8 ±0.8 spots per nurse cell nucleus; p < 2.3x10^−10^, *CK1α^G0492^* */+; SMC4^k00819^*/+; *Cap-H2^z3–0019^*/+: 2L = 11.4 ±0.6; p < 1.2x10^−14^) (Fig. 5F-G and S5D–E Fig.). However, these additional mutants did not suppress the unpairing defect as well as the *CK1α^8B12^* */+* mutant. The *CK1α^EP1555^* and *CK1α^G0492^* alleles were only able to rescue the condensin II unpairing defect to levels approximately 50% of the control flies. It is possible that these two additional mutants, *CK1α^EP1555^* */+* and the *CK1α^G0492^* */+*, are not complete protein nulls, like the *CK1α^8B12^* mutation. Nevertheless, three different and independently derived alleles of *CK1α* suppress the condensin II unpairing defect, providing strong genetic support for CK1α functioning as a negative regulator of Cap-H2 in vivo. These observations are also consistent with CK1α RNAi depletion in cultured cells resulting in hyper-activation of Cap-H2 chromosome anti-pairing function (Fig. 3H,M,N).

### CK1α limits chromatin bound Cap-H2 levels in salivary glands

**Fig 6:**
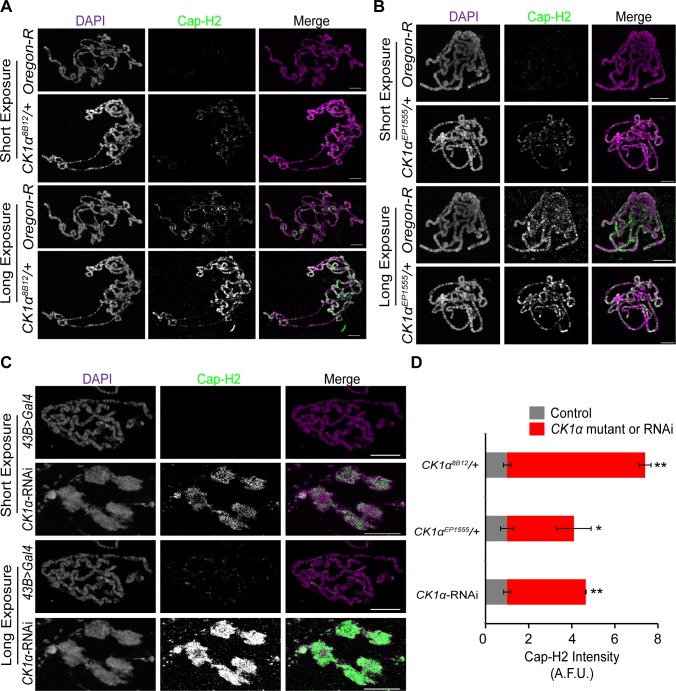
CK1α limits chromatin bound Cap-H2 levels and activity in *Drosophila* salivary glands. (A-B) Micrographs of salivary glands from larvae of wild-type (Oregon-R) and CK1α heterozygous mutants (*CK1α^8B12^* */+* and *CK1α* ^EP1555^ */+*) squashed, immunostained for Cap-H2 (green) and counterstained for DNA (DAPI, magenta) on the same glass microscope slide. (C) Micrographs of salivary glands from *Drosophila* larvae of control (*43B>Gal4*) and larvae expressing RNAi to CK1α (*CK1α^RNAi^*) squashed, immunostained for Cap-H2 (green) and counterstained for DNA (DAPI, magenta) on the same glass microscope slide. Short and long camera exposures were used in order to show that chromatin bound Cap-H2 levels are increased in both CK1α mutants and CK1α RNAi depleted cells. (C) CK1α RNAi resulted in loss of polytene banding and suggests that chromosomes are unpaired (see Fig. 4). (D) Histogram showing quantitation of fluorescence intensity of Cap-H2 bound to squashed salivary gland chromosomes. CK1α reduction via mutation or RNAi results in significant enrichment of Cap-H2 over same slide controls, assessed by comparing normalized Cap-H2 fluorescence (gray value) intensities (see methods for details); (n = 5–6 fields of squashed chromosomes per genotype). Images shown were not used for analysis. Images used for fluorescence intensity quantitation were acquired such that pixel saturation was minimized. Error bars indicate SEM. p-value = * < 0.05, ** < 0.0001 (calculated by using students’ t-test in Microscoft excel). Statistical comparisons are between mutant or RNAi depleted cells and their control on the same slide (mutant control = *OregonR*; RNAi control = *43B>Gal4*). Maximum projection images of multiple z-slices are shown for all panels. Scale, 20μm in all panels.

A possible mechanism for CK1α regulation of condensin II activity may be by maintaining low levels of chromatin bound Cap-H2. To test whether chromatin bound Cap-H2 is elevated in response to reduction of CK1α levels, we performed immunostaining on polytene chromosomes from salivary glands of third instar larvae. Maintaining a low level of Cap-H2 in this tissue is necessary for the maintenance of the highly paired state of polytene chromosomes, as overexpression of Cap-H2 can drive these chromosomes to become highly unpaired[16]. Salivary glands were dissected from wild type control and CK1α heterozygous mutants, squashed onto microscope slides, and immunostained for Cap-H2 to assess levels of endogenous chromatin bound protein. Two different CK1α mutants, *CK1α^8B12^* and *CK1α^EP1555^* were used (as in Fig. 5), each containing an independently derived disruption of the *CK1α* gene. Salivary glands from wild-type (*Oregon R*) and CK1α mutants were squashed onto the same microscope slide such that all chromosome spreads simultaneously received the same mixture of washes and antibody reagents, as previously described [43]. This approach allowed us to use identical imaging parameters to make comparisons between different genotypes from the same slide. In wild type (*Oregon-R*) salivary glands, Cap-H2 is present on the chromatin at low levels (Fig. 6A-B) and requires a higher exposure to detect the protein signal. However, salivary glands from mutants with either of the two heterozygous CK1α mutations show a visibly higher level of Cap-H2 on the chromatin, which is apparent even at the lower exposure, demonstrating that chromatin bound Cap-H2 protein levels are elevated (Fig. 6A-B). Quantitation of fluorescence intensity confirms that Cap-H2 protein levels on the salivary gland chromosomes is significantly higher in CK1α heterozygous mutants (*CK1α^8B12^*/+: p < 1.8x10^−7^, *CK1α^EP1555^*/+: p < 0.05 when compared to wild-type controls) (Fig. 6D). These results suggest that CK1α is required to maintain low levels of chromatin bound Cap-H2, as half dosage CK1α mutation is sufficient to increase chromatin bound Cap-H2.

In addition to the CK1α heterozygous mutants, we performed immunostaining for Cap-H2 in flies expressing CK1α RNAi under a UAS-regulated promoter crossed with a *43B>Gal4* transgene, a salivary gland specific driver [44]. Similar to the results seen with the CK1α heterozygous mutants, CK1α RNAi resulted in a visible increase in chromatin bound Cap-H2 (Fig. 6C). The significant increase (p < 4.4x10^−5^) in Cap-H2 fluorescence intensity confirms this observation (Fig. 6D). In addition to the increased level of Cap-H2 staining on the DNA, we also noticed chromosome aberrations, where the polytene banding pattern was clearly disrupted. These abnormal chromosome structures are reminiscent of the unpaired chromosomes seen in whole mount salivary gland nuclei that are overexpressing Cap-H2 (Fig. 4) [16,22]. The abnormal chromosomes also display a higher intensity of Cap-H2 staining (Fig. 6D) and further support the idea that an increase in Cap-H2 levels via CK1α depletion is driving the chromosomes to become highly unpaired.

### Casein Kinase I Alpha is required for Cap-H2 degradation

**Fig 7:**
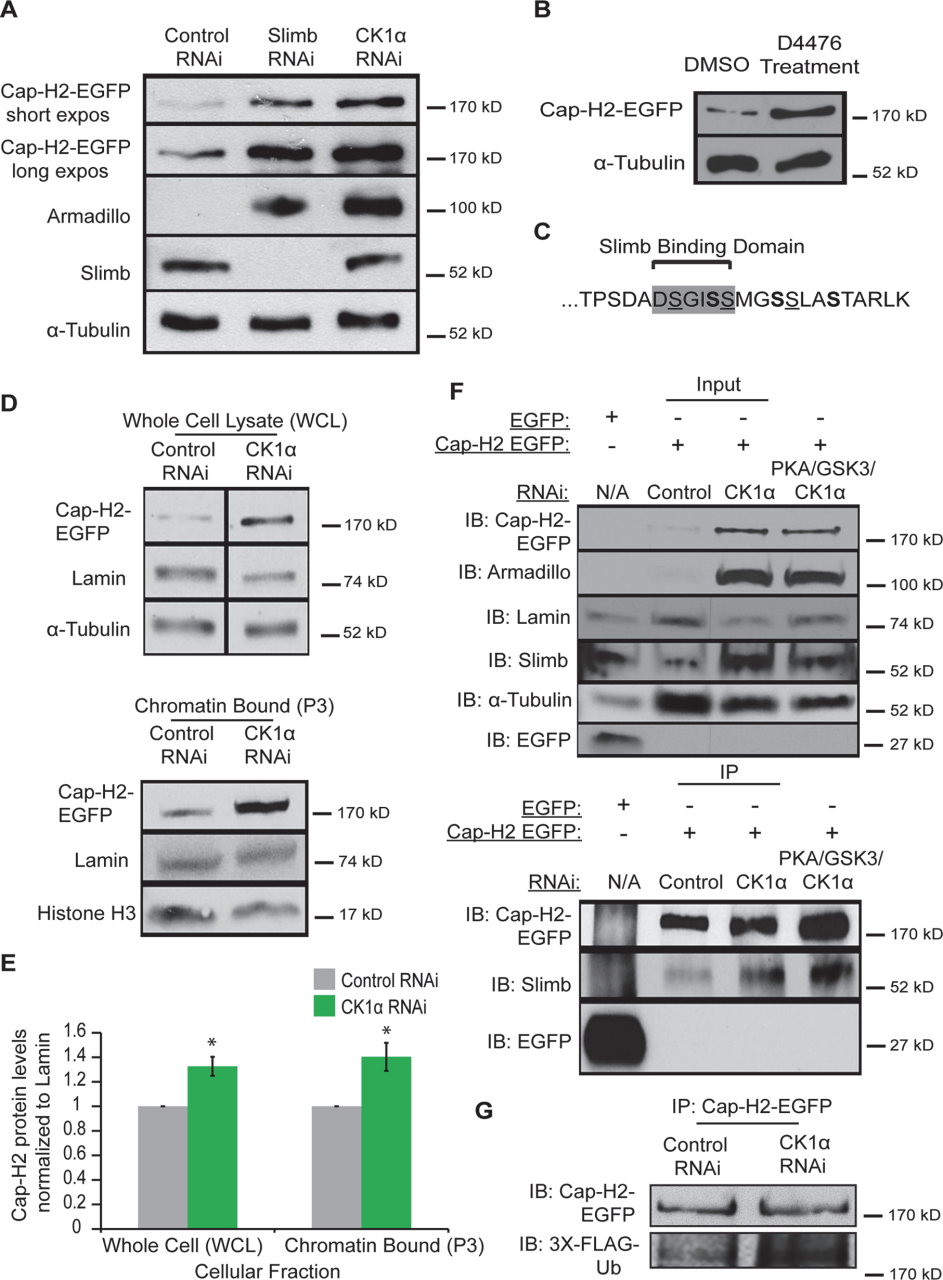
Cap-H2 degradation is CK1α dependent. (A) Whole cell lysates from RNAi treated S2 stable line expressing an inducible Cap-H2-EGFP were immunoblotted for the indicated proteins. Both Slimb and CK1α depletion stabilizes Cap-H2-EGFP. Armadillo is targeted for destruction by Slimb and CK1α and is used to confirm knockdown of CK1α and Slimb. (B) Kc cells were transiently transfected with a plasmid containing an inducible Cap-H2-EGFP, and then treated with either DMSO (control) or 80μM of CK1 inhibitor (D4476) for 8 hours, and lysates were immunoblotted for the indicated proteins. Anti-alpha-Tubulin was used as protein loading control in panels A and B. (C) Amino acid sequence of C-terminal 23 amino acids of Drosophila Cap-H2. Consensus Slimb binding domain DSGISS highlighted in grey. This contains three S-X-X-**S** motifs, where the underlined serines denote potential priming sites and bold serines indicate potential CK1α phosphorylation sites and X can be any amino acid. (D) Cellular fractions of RNAi treated Kc stable line expressing an inducible Cap-H2-EGFP immunoblotted for the indicated proteins. CK1α depletion stabilizes Cap-H2-EGFP in both the whole cell lysate (WCL, top immunoblot) and the chromatin bound (P3, bottom immunoblot) cellular fractions. Anti-alpha-Tubulin was used as loading control for WCL, Anti-Histone-H3 was used as loading control for P3, and Anti-Lamin-Dm0 was used as loading control for both WCL and P3. (E) Fold enrichment of Cap-H2-EGFP protein levels from (D). CK1α depletion stabilizes whole cell (WCL) and chromatin bound (P3) Cap-H2-EGFP protein levels when normalized to Lamin. Calculated using densitometry in ImageJ. Error bars indicate SEM. p-value = * < 0.05, (calculated by using students’ t-test in Microscoft excel). (n = 4 biological replicates). (F) Immunoprecipitations and immunoblots from RNAi treated Kc cells, transiently transfected with inducible EGFP as a negative control and Kc cells stably expressing an inducible Cap-H2-EGFP. Anti-Cap-H2-EGFP immunoprecipitates Slimb in control, CK1α, and PKA/GSK3β/CK1α depleted cells expressing Cap-H2-EGFP. GFP tag only transfected cells did not immunoprecipitate Slimb. Anti-armadillo was used to verify CK1α and PKA/GSK3β/CK1α depletion and anti-Lamin-Dm0 and anti-alpha-tubulin was used as protein loading control. (G) Immunoprecipitations and immunoblots from RNAi trated S2 cells, transiently co-transfected with inducible Cap-H2-EGFP and inducible 3X-Flag-Ubiquitin. Anti-Cap-H2-EGFP immunoprecipitates 3X-Flag-Ub in both control and CK1α depleted cells expressing Cap-H2-EGFP.

Depletion of CK1α resulted in increased condensin II activity and chromatin bound Cap-H2 levels. We next wanted to test if CK1α is functioning to degrade Cap-H2 protein. In order to do this, we assayed Cap-H2 levels in cultured cells treated with CK1α RNAi. In order to functionally validate depletion of CK1α by RNAi, the level of the Slimb and CK1α substrate Armadillo was determined by immunochemistry [32,34]. Slimb and CK1α normally function to repress Armadillo protein levels. RNAi depletion of CK1α resulted in stabilization of Armadillo to levels higher than that of Slimb depletion, confirming efficient depletion of CK1α (Fig. 7A). Whole cell extracts from cultured S2 cells stably expressing an inducible Cap-H2-EGFP depleted of CK1α by RNAi showed stabilization of EGFP tagged Cap-H2 as compared to control treated cells (Fig. 7A). In addition, Kc cells treated with the CK1 inhibitor D4476 also resulted in stabilization of a transiently transfected and induced Cap-H2-GFP protein (Fig. 7B). These results further support the idea that CK1α negatively regulates condensin II through its subunit, Cap-H2, as depletion or inhibition of CK1α results in stabilization of Cap-H2 protein.

Based on the results of stabilization of Cap-H2 when CK1α is inhibited in cultured cells and the increase in Cap-H2 fluorescence on CK1α mutant salivary gland chromosomes (Figs. 6 and 7A-B), we hypothesized that a normal function of CK1α may be to limit chromatin bound Cap-H2 levels. In order to test this, we performed cellular fractionations to ask if chromatin bound Cap-H2 levels were stabilized in the absence of CK1α. For these experiments, we used a Kc cell line that stably expresses an inducible Cap-H2-EGFP and treated them as before with RNAi. CK1α-RNAi depletion resulted in an increase of chromatin bound Cap-H2-EGFP by 40 ±0.11%, as compared to chromatin extracts from control-RNAi treated cells (Fig. 7D-E). This result demonstrates that CK1α is negatively modulating both whole cell and more specifically, chromatin bound Cap-H2 protein levels. More importantly, this suggests that CK1α normally inhibits condensin II acitivity in part by limiting the levels of chromatin bound Cap-H2 protein.

### CK1α depletion does not affect Slimb and Cap-H2 interaction

We previously demonstrated that Slimb and Cap-H2 proteins co-immunoprecipitate [20]. Based on reports of how CK1α is required for Slimb interaction with its targets, for example the Beta-Catenin/Armadillo, Ci and Cdc25 proteins[34,45,46], we speculated that CK1α is also required for Slimb interacting with Cap-H2 and promoting Cap-H2 ubiquitination. To test this, we performed immunoprecipitations in CK1α-RNAi depleted Kc cells that stably expressed an inducible Cap-H2-EGFP. To our surprise, when Cap-H2-EGFP was immunoprecipitated, endogenous Slimb also co-immunoprecipitated in both control-RNAi treated and CK1α-RNAi treated cells (Fig. 7F). Slimb did not co-immunoprecipitate with EGFP tag only controls transiently transfected into untreated Kc cells. Similar results were observed in S2 cells transiently transfected with GFP tag only and Cap-H2-EGFP (S6 Fig.). The unexpected result of CK1α depletion having no effect on Cap-H2 and Slimb interaction led us to postulate that an additional kinase(s) may be functioning upstream of CK1α to prime Cap-H2, permitting Slimb interaction. We decided to perform immunoprecipitations in Kc stable cells that were codepleted of PKA, GSK3β, and CK1α. As discussed earlier, PKA and GSK3β have been shown to work with CK1α to phosphorylate Slimb substrates. Surprisingly, Cap-H2-EGFP and Slimb interaction remained unperturbed in the PKA, GSK3β, and CK1α co-depleted cells (Fig. 7F). Furthermore, to see if Cap-H2 was still being ubiquitinated in CK1α depleted cells, immunoprecipitations were performed in RNAi treated S2 cells co-transfected with Cap-H2-EGFP and 3xFLAG-tagged ubiquitin. In both control-RNAi and CK1α-RNAi depleted cells, immunoprecipitated Cap-H2-EGFP was found to be labeled with FLAG-ubiquitin (Fig. 7G). These observations are surprising because Slimb interaction with its protein targets is thought to result in target-protein proteolysis. However, here we observe that although both CK1α and Slimb are required for targeting Cap-H2 for degradation, Slimb can still interact with Cap-H2 when stabilized by CK1α depletion. We speculate on possible explanations in the discussion.

## Discussion

Drosophila condensin II functions in interphase genome organization through its role in regulating chromosome compaction, homolog pairing and dispersal of centromeres [16,18,19,20,21,22]. In this study, we report a previously unidentified role of Casein Kinase I Alpha (CK1α). We find that CK1α is an important modulator of interphase genome organization, regulating homologous chromosome pairing, centromere clustering, and chromosome compaction in Drosophila cultured cells and in vivo. Furthermore, we show that CK1α affects these processes by attenuating interphase condensin II activity as CK1α function is required for protein turn-over of the Cap-H2 condensin II subunit.

Using an RNAi based approach in Drosophila cultured cells, we observed that depletion of CK1α resulted in perturbation of interphase nuclear morphology (Fig. 1). We also find that CK1α functions to prevent centromere dispersal (Fig. 2 and S2 Fig.), inhibits chromosome compaction (Fig. 3), and promotes chromosome pairing (Figs. 3,4,5 and S2 and S5 Figs.). These observations are consistent with the changes seen when Cap-H2 is overexpressed. In cultured Drosophila cells, co-depletion of CK1α with the condensin II subunit Cap-H2 results in suppression of abnormal centromere dispersal, suppression of chromosome hyper-compaction, and suppression of chromosome unpairing (Figs. 1,2,3 and S2 Fig.). These observations strongly suggest that CK1α and Cap-H2 interact genetically. This interaction is also observed in vivo, as the Drosophila nurse cell chromosome unpairing defect seen in condensin II mutants (*SMC4^k00819^* */+; Cap-H2^z30019^* */+*) is suppressed by three independent CK1α heterozygous mutations (Fig. 5 and S5 Fig.). Furthermore, CK1α-RNAi depletion or mutations increases chromatin bound Cap-H2 protein levels (Figs. 6 and 7). This was determined by immunofluorescence of endogenous Cap-H2 protein on polytene chromosomes as well as by sub-cellular fractionation of chromatin bound proteins from Cap-H2-EGFP expressing cells in culture. It is important to emphasize that Cap-H2 and other condensin II gene loss-of-function mutations do have oogenesis phenotypes that are completely suppressed by decreasing the dosage of CK1α by half (Fig. 5 and S5 Fig.). Together, these findings demonstrate CK1α as a novel regulator of interphase condensin II levels and activity.

CK1α is a highly conserved serine/threonine kinase involved in Wnt signaling pathways, DNA repair, cell cycle progression, and mRNA metabolism [35,47,48]. Identification of CK1α furthers our understanding of the mechanisms by which condensin II is regulated. The chromodomain protein Mrg15 is involved in the loading of Cap-H2, while the E3 Ubiquitin ligase, SCF^Slimb^ ubiquitylates Cap-H2, removing it from chromatin and targeting it for proteasomal degradation [20,21]. Phosphorylation is known to be a prerequisite for Slimb recognition of its target proteins [29,49]. CK1α has been shown to require other kinases that prime its recognition site. For example, CK1α can phosphorylate the second S/T in the S/T-X-X-S/T motif but only when the S/T in the first position is already phosphorylated by a different kinase [33,50]. Interestingly, the C-terminal end of Drosophila Cap-H2, TPSDADSGI**S** SMG**S** SLA**S**TARLK, contains three S-X-X-S sites, where the underlined serines denote potential priming sites and bold serines indicate potential CK1α phosphorylation sites (Fig. 7C). However, our efforts to test a direct interaction between CK1α and Cap-H2 were unsuccessful, as we were unable to produce kinase-active CK1α to determine whether CK1α can phosphorylate recombinant Cap-H2 in vitro. Because depletion of CK1α results in stabilization of Cap-H2 protein and increased condensin II activity (Figs. 6 and 7), we speculate that Cap-H2 itself is targeted by CK1α but likely requires an additional kinase to prime Cap-H2 phosphorylation, as previously shown for other substrates like Armadillo/β-Catenin [33,50]. This hypothesis is further supported by the observation that the Cap-H2-Slimb interaction persists and that Cap-H2 is still ubiquitinated when CK1α is depleted (Fig. 7F-G and S6 Fig.). It can be speculated that the initial steps of Cap-H2 degradation involve an additional regulator, perhaps a kinase priming Cap-H2 at specific residues, permitting Slimb interaction and ubiquitination of Cap-H2 at specific sites. However, the final step for Cap-H2 targeting for degradation and removal from chromatin may be mediated by CK1α. Alternatively, it is possible that CK1α is indirectly regulating Cap-H2, perhaps through an intermediate. CK1α could be phosphorylating a regulator of Cap-H2 which determines Cap-H2 protein levels. For example, it is possible that although Slimb binding to Cap-H2 is independent of CK1α, this kinase may phosphorylate some other protein or Slimb itself before the Cap-H2 protein can be fully degraded. Clearly how Slimb and CK1α regulate Cap-H2 levels is more complex than previously appreciated. A recent study has shown that Armadillo/β-Catenin is protected from degradation by the Armless protein binding to and inhibiting Ter95, a component of the SCF^Slimb^ E3-ligase[51]. This raises the possibility that Cap-H2 may similarly be protected from proteolysis, and Slimb-bound Cap-H2 molecules may require CK1α to eliminate this protective function. While we have not been able to show a direct interaction between CK1α and Cap-H2, it is clear from our experiments in cultured cells and in Drosophila tissues that CK1α limits Cap-H2 protein levels, thereby attenuating interphase condensin II activity.

**Fig 8:**
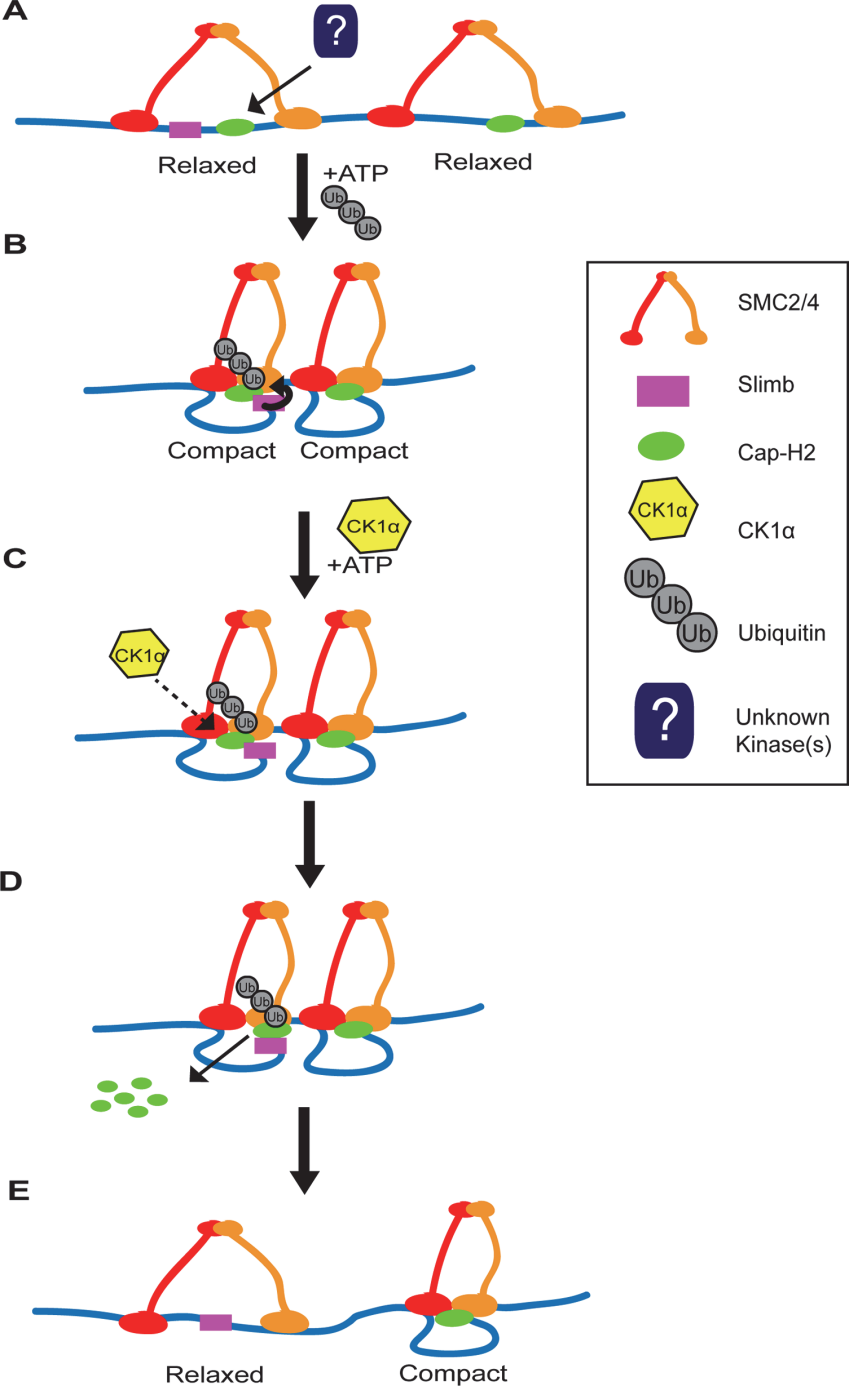
Two-step model of Cap-H2 eviction from chromatin. (A) SMC dimers (red and orange) are associated with DNA (blue line) in the “open” conformation, resulting in “relaxed” chromatin. There may be regions where Cap-H2 (green oval) is bound with or without Slimb (magenta square) nearby. (B) ATP binding to SMC dimers promotes the “closing” of the SMC subunits, resulting in the “compaction” of chromatin. Cap-H2 binding to SMC dimers traps a closed SMC2/4 dimer and inhibits ATP hydrolysis. Inhibition of ATP hydrolysis by Cap-H2 is thought to maintain SMC dimers in the “closed” conformation and promotes axial chromosome compaction. (C) CK1α negatively regulates Cap-H2. This may occur by directly phosphorylating Cap-H2 or indirectly by CK1α targeting an unknown regulator of Cap-H2. (D) CK1α promotes Cap-H2 removal from chromatin and degradation. Note that Slimb interaction with Cap-H2 is not likely to be dependent on CK1α activity. In chromatin regions where Slimb and CK1α are absent (condensin II complex on the right), Cap-H2 could remain on the chromatin, as it avoids Slimb and CK1α mediated degradation. (E) Cap-H2 removal from chromatin results in the opening of the SMC dimer, resulting in the “relaxation” of chromatin (left side), whereas on chromatin where Slimb is absent, Cap-H2 remains chromatin bound and the chromatin remains axially compact (right side).

Having multiple regulators of Cap-H2 allows for precise modulation of condensin II activity. We have previously shown that both Cap-H2 and Slimb are chromatin bound [20]. Whether CK1α is also bound to chromatin is unknown. CK1α associates with centrosomes in Chinese hamster ovary cells and CK1α enters the nucleus after DNA damage induction in Drosophila embryos [35,52]. If CK1α interaction with Cap-H2 precedes and is required for Slimb mediated Cap-H2 turnover, having a two-step requirement for Cap-H2 degradation would allow for more precise control over the spatial regulation of condensin II activity. We speculate that at chromatin regions where both Slimb and Cap-H2 are localized, condensin II activity can remain high while Cap-H2 remains dephosphorylated within the Slimb recognition site (Fig. 8). However, condensin II activity can be reduced quickly by localizing CK1α to these specific regions and/or by regulated nuclear import of CK1α[35], resulting in destruction of Cap-H2 protein. This speculative model of Cap-H2 removal from chromatin is attractive in that it would allow for a “switch-like” change in condensin activity that is responsive to developmental cues and environmental stressors. Furthermore, by differential distribution of Cap-H2 loading factors like Mrg15[21] and inhibitors like Slimb [18,20] this model would allow for precise changes in chromosome morphology at the local chromatin level, possibly giving rise to local regions of high and low compaction that may define structures such as topologically associated domains (TADs).

It should be noted that there are four predicted Drosophila Cap-H2 splice isoforms. One of these isoforms, Cap-H2-RD, lacks a region of 64 amino acids in the C-terminus, present in the other three isoforms. This raises the possibility that Cap-H2-RD may be resistant to Slimb ubiquitination. We speculate that this isoform could provide areas of constitutive condensin II activity, where the chromatin is unpaired and/or providing local regions of high compaction. In Drosophila it is thought that the genome exhibits region-specific levels of homolog pairing, for example heterochromatin and euchromatin exhibit different somatic pairing properties [18,38,53]. Having different Cap-H2 isoforms and differential localization of Cap-H2 regulators bound to specific regions of chromatin could provide the necessary landscape to enable differences in regional pairing and compaction state, while regulators such as Slimb and CK1α add plasticity to compaction states by modulating chromatin bound Cap-H2 levels.

Condensin II modulation of interphase chromatin compaction has also been shown to affect accessibility of other chromatin proteins. It was observed in Cap-H2 mutant mice, that loss of Cap-H2 resulted in disruption of T-cell differentiation [54,55]. Interestingly, chromatin condensation is involved in mouse T-cell differentiation. In response to cytokine signaling, naïve T-cell chromatin must decondense in order to allow for STAT5 transcription factor binding. Furthermore, this decondensation is dependent on the inactivation of condensin II [27]. Clearly, limiting interphase condensin II activity is important in this context, and it remains to be determined what mechanism is acting to repress condensin II function during mammalian T cell activation. It is tempting to speculate that cytokine signaling could trigger the activation of a condensin II antagonist, leading to the decrease in condensin II activity. This would lead to decondensation of chromatin allowing STAT5 access to DNA. Our findings in the Drosophila model suggest that similar interphase condensin II functions may be at play, and CK1α along with Slimb are critical regulators of this condensin II activity. However, at present it is not known if mammalian condensin II activity is regulated by Slimb or CK1α, and it should be noted that mouse and human Cap-H2 do not have clear Slimb binding consensus sequences. It will be of great value to identify additional kinases that may collaborate with CK1α and Slimb to negatively regulate Drosophila condensin II activity, and to further elucidate the biological significance of this interphase condensin II function in Drosophila and other species.

## Materials and Methods

### Cell culture and double-stranded RNAi

*Drosophila* cell culture, in vitro dsRNA synthesis, and RNAi treatments were performed as previously described [56]. S2 and Kc cells were cultured in Sf900-II media (Life Technologies) supplemented with 1X Antibiotic-Antimycotic (Life Technologies). S2R+ cells were cultured in Schneider’s media (Life Technologies) supplemented with 1X Penicillin Streptomycin (Corning Cellgro) and 10% Fetal Bovine Serum (Thermo Scientific; Hyclone). RNAi treatments were performed in 6-well tissue culture plates, with 10μg of dsRNA in 1mL media administered to confluent (50–90%) wells. Wells were replenished with fresh 1mL of media and 10μg dsRNA every other day for 4–7 days. dsRNA was made using gene specific primer sequences listed in S1 Table. Control (SK) dsRNA was made by amplifying off PCR product from a non-GFP sequence of the pEGFP-N1 vector (Takara Bio Inc.). PCR products were then used as template for dsRNA production using T7 RiboMAX Express Large Scale RNA Production System kit (Promega, # P1320). dsRNA concentration was then calculated using gel electrophoresis and densitometry analysis (NIH ImageJ).

Cell viability experiments were performed with Kc cells treated with dsRNA and cultured as described above with modifications. 750,000 cells per well were plated in triplicates into a 24-well tissue culture plate (Falcon) with 300μL of media and 3μg of dsRNA per well and treated every other day for 4–6 days. At days 4 and 6, aliquots of cells were taken for Propidum Iodide and DAPI staining for cell viability analysis. Approximately one third (100μL) of each well was taken and 150μL new media was added containing Propidium Iodide (1ng/μL) and DAPI (2μg/mL) and incubated at 25°c for 30 minutes. Mixture was then spun down and resuspended in 10μL new media and plated onto microscope slides and coverslips to image. Image fields were obtained and used for counting with nuclei co-staining for both Propidium Iodide and DAPI being counted as a “dead” cell.

### Constructs and transfections

Construct production and transfection were performed as described previously [20]. cDNA encoding Cap-H2-EGFP or *Drosophila* ubiquitin (3x-Flag-Ubiquitin) were subcloned into the inducible metallothionein promoter pMT vector, as previously described[20]. Transient transfections were performed using the Nucleofector II (Lonza) according to manufacturer’s instructions. Transiently transfected cells (Fig. 7B,F,G) were induced 24 hours post transfection. Stable S2 cell lines (Fig. 7A) were selected by co-transfection with pCoHygro (Life Technologies) plasmid and treated for 3–4 weeks with Hygromycin B (Life Technologies). Expression of all constructs was induced by addition of 1mM CuSO_4_ to the media and processed for downstream experiments 24 hours later. Transiently transfected Kc cells in Fig. 7B were cotransfected with pMT-eGFP and pMT-Cap-H2-eGFP in order to confirm equal transfection efficiency between treatments. Stable Kc cell lines under inducible metallothionein promoter (Fig. 7D) were provided by the Wu lab (Harvard Medical School).

### CK1α drug inhibition

D4476, chemical Casein Kinase I inhibitor (EMD Millipore Calbiochem) was resuspended in DMSO to 50μM stock dilutions. D4476 was then diluted into cell culture media at 80μM and added to cells (60–95% confluent) in 6-well tissue culture plates. Cells were treated for 8 hours and prepped for immunoblotting or plated onto Concanavalin-A (Sigma-Aldrich) coated coverslips for immunostaining, as previously described. Control treated cells were treated with same volume of DMSO under same conditions as drug treatment.

### Immunofluorescence

Cultured cells were immunostained as previously described [56]. Cells were plated onto Concanavalin-A (Sigma-Aldrich) coated cover slips, allowed to adhere onto coverslips for 20min then fixed with 10% formaldehyde (Ted Pella, INC) in PBS at room temperature. Cells were then washed with PBS and then 0.1% PBS/Triton to permeabilize. Cells were then blocked with Block Solution (5% normal goat serum (Sigma-Aldrich), 0.1% PBS/Triton, and 1mM Sodium Azide (Sigma-Aldrich)) for 1 hour at room temperature prior to immunostaining. Primary antibodies were diluted in block solution and coverslips were incubated with primary antibody for 1 hour at room temperature. Primary antibody concentrations used were as follows: rabbit anti-CID at 1:400 [20], mouse anti-Lamin (Dm_0_) ADL 84.12 at 1:200 (*Drosophila* Hybridoma Bank, University of Iowa), rabbit anti-phosphorylated-Histone-H3 at 1:500 (Upstate; EMD Millipore), and rabbit anti-Cap-H2 at 1:50 [16]. Cells were then washed with 0.1% PBS/Triton three times for 5 minutes each at RT. Secondary antibodies (conjugated to Alexa 488 (Life Technologies), Cy-2, or Cy-3 (Jackson ImmunoResearch Laboratories) were diluted in block solution and coverslips were incubated with secondary for 2 hours at room temperature. Secondary antibody concentrations used were as follows: Alexa 488 at 1:500, Cy-2 at 1:500, and Cy-3 at 1:500. Coverslips were then washed with 0.1% PBS/Triton three times for 5 minutes each at RT. Coverslips were then washed with PBS for 5 minutes at RT. 4’,6-Diamidino-2-Phenylindole, Dihydrochloride (DAPI) (Life Technologies) was used at a final concentration of 1μg/mL in PBS for 10 minutes to stain DNA. Coverslips were then washed two times in PBS for 5 minutes. Coverslips were then mounted onto microscope slides and were mounted using Vectashield (Vector Labs) and sealed with nail polish and stored at -20 degrees.

Whole mount ovaries were immunostained as previously described [21]. Tissues were dissected in PBS and fixed in 4% formaldehyde/PBS for 10 minutes. Tissues were then rinsed with 0.1% PBS/Triton and blocked with block solution (same as used for cell staining) for 30 minutes. Tissues were incubated with primary antibodies (same as above) overnight at 4° rotating on rotator. Tissue was then washed in 0.1% PBS/Triton three times for 5 minutes while rotating at room temperature. Tissues were then blocked two times for 15 minutes while rotating at room temperature. Secondary antibodies (same as above) were incubated with samples for 2 hours. Tissues were rinsed with 0.1% PBS/Triton then PBS and counterstained with DAPI for 10 minutes. Tissues were then mounted onto slides with Vectashield and sealed using nail polish.

Salivary gland squashes were performed as previously described [57] with modifications. Salivary glands were dissected in 0.7% NaCl and were placed onto Repel silane (GE Healthcare) coated cover slips and fixed in 3.7% formaldehyde in 45% acetic acid for 2.5 minutes. Coverslips with glands were then inverted onto a glass poly-L-lysine (Sigma) coated microscope slide and squashed with an orthopedic hammer until nuclei burst. Glands from different genotypes were squashed onto different sections of the same slide to serve as an internal control. The location of the different glands was noted on the slide, as previously described [43]. Slides/coverslips were then dipped into liquid nitrogen and coverslips were removed using a razor blade. Squashed chromosomes were then washed once in PBS at RT. Antibodies (same as above) were diluted in PBS, 0.1% NP40 (Sigma-Aldrich), and 1% non-fat milk (Carnation) and added to slide and covered with cover slip. Primary antibody incubations were performed in humid chamber at 4 degrees overnight. Slides were then washed in 0.1% NP40/PBS for 5 minutes and secondary antibodies (same as above) were added and covered with cover slip for 2 hours in humid chamber at RT. Slides were then washed three times in PBS and counterstained with DAPI (1μg/mL) for 25 seconds and washed for 5 minutes in PBS. Slides were mounted with coverslip and Vectashield and sealed with nail polish.

### Fluorescent in situ hybridization (FISH)

Whole mount salivary gland and ovary FISH was performed by dissecting tissues into PBS. Tissues were then fixed in 100 mM sodium cacodylate, 100 mM sucrose, 40 mM sodium acetate, 10 mM EGTA, and 3.7% formaldehyde for 4 min at RT. Tissues were then rinsed in 2X SSCT (0.3 M NaCl, 0.03 M sodium citrate, pH 7.0, and 0.1% Triton) and treated with 2μg/mL Ribonuclease A (Sigma-Aldrich) for 1 hour. 10 minute stepwise washes were performed in 20%, 40%, and 50% formamide (Sigma-Aldrich) in 2X SSCT. Tissues were pre-hybridized in 50% formamide/2X SSCT for 2 hours in 37° water bath. 1–2 μl of each probe in hybridization solution (Dextran sulfate (Sigma-Aldrich), NaCl, sodium citrate, formamide, H_2_O) to a total volume of 40 μL was added to 0.2 mL PCR tube with tissues. Hybridization was performed using a thermal cycler, 91° for 2 minutes to denature and 37° overnight for hybridization. Tissues were then washed in 50% formamide in 2X SSCT three times for 30 minutes. 10 minute stepwise washes were performed in 40% then 20% formamide in 2X SSCT. Tissues were then washed three times in 2X SSCT for 10 minutes, 10 minutes in 0.1% PBS/Triton, and then counterstained with DAPI (1 μg/mL) in PBS for 10 minutes at RT. Two 10 minute PBS washes were performed and tissues were mounted in Vectashield and sealed with nail polish.

Cultured cell FISH was performed as previously described [20]. Cells were plated onto Con-A coated coverslips in a well of a 6-well tissue culture plate for 20 minutes and allowed to adhere to coverslips at room temperature. Coverslips were then washed with 1X PBS and fixed in 10% Formaldehyde/PBS for 10 minutes at RT. Coverslips were then washed once with PBS and permeabalized in 0.1% PBS/Triton for 10 minutes. Cells were then washed in CSK buffer (10mM HEPES, 100mM NaCl, 3mM MgCl_2_, 300 mM Sucrose, and Phenylmethanesufonyl fluoride (PMSF)) for 10 minutes and Ribonuclease A (100ug/mL) for 1 hour at RT. Cells were then washed with 0.1N HCl for 5 minutes and taken through ethanol series for 5 minutes each (70%, 90%, then 100%). One 2X SSCT wash was performed and cells were pre-hybed in 50% formamide/2X SSCT at 37 degrees for 2 hours. FISH probe (1–2 μL each probe) was then added to hybridization solution (total 25 μL) and this mixture was denatured at 95° for 2 minutes and snap cooled in ice bath. Probe mixture was then added onto microscope slide and coverslips were inverted onto the microscope slide. Coverslips were then sealed with rubber cement and slide/coverslip was denatured at 93° on a heat block for 3 minutes. Slides were then placed in humid chamber and hybridized overnight at 37°. After hybridization was complete, coverslips were detached from slides by immersing in 50% formamide/2X SSCT with shaking for 10 minutes. Coverslips were then placed into 6-well tissue culture plates and washed three times for 30 minutes at 42° (all heterochromatin probe post washes performed at 37°). Ten minute washes at 42° were then performed with 40% then 20% formamide in 2X SSCT. Three 2X SSCT washes were performed for 5 minutes each at RT on shaker. Cells were then counterstained with DAPI (1μg/mL) in PBS for 10 minutes at RT. Coverslips were then washed two times for 10 minutes at RT in PBS. Cells were then mounted with Vectashield and sealed with nail polish.

### FISH probe preparation

Euchromatic FISH probes were made as previously described [20,21] from BAC clones (Children’s Hospital Oakland Research Institute BACPAC Resources) as follows: X1, BACR30C13 and BACR18F10; X2, BACR20K01 and BACR35A18; X3, BACR11C13 and BACR07F15; 2L (1) BACR30M19 and BACR29P12. For nurse cell and salivary gland FISH: X, BACR20K01 and BACR35A18; 2L, BACR30M19 and BACR29P12. BAC clones were mapped and picked by using the UCSC genome browser (http://www.genome.ucsd.edu). BAC clones were cultured and DNA was purified using QIAGEN Plasmid Midi Kit (Qiagen 12143). Purified BAC DNA was amplified using Whole genome amplification kit (Sigma-Aldrich WGA1), 20 μg of amplified DNA was then digested using a cocktail of AluI, Rsa, MseI, MspI, HaeIII, and BfuCl (New England BioLabs) overnight at 37°, ethanol precipitated, and resuspended in 36 μL ddH_2_O. DNA was denatured at 100°C for 1 min, and then 3'-end-labeled with unmodified aminoallyl dUTP and terminal deoxynucleotidyl transferase (Roche). After incubating for 2 h at 37°, 5 mM EDTA was added to terminate the reaction. DNA was ethanol precipitated, resuspended in 10uL ddH2O, and then conjugated to fluorophores using ARES Alexa Fluor DNA labeling kits (A-21665, A21667, and A-21676; Life Technologies) for 2 h, according to the manufacturer’s instructions. Probes were then cleaned up using Qiagen PCR clean up kit (Qiagen), ethanol precipitated, and resuspended in 10μL EB buffer (Qiagen).

Heterochromatic FISH probes were made using 10 μg of oligonucleotide primers (Integrated DNA Technologies) diluted in TE buffer using sequences: for heterochromatin to 2R: 5'-AACACAACACAACACAACACAACACAACAC-3', for heterochromatin to 3R: 5'-CCCGTACTGGTCCCGTACTGGTCCCGTACTCGGTCCCGTACTCGGT-3'. DNA was denatured at 100° for 1 minute and snap cooled on ice, DNA was then end labeled using 3’-end labeling with fluorophore conjugated dUTP (cy3 and cy5) (GE Healthcare Amersham) using terminal deoxynucleotidyl transferase (Roche). Reactions were left in 37° water bath for 2 hours. Reactions were stopped by adding 1 μL of 0.25 mM EDTA. Probes were then ethanol precipitated and resuspended in TE buffer.

### Microscopy/Image analysis

Micrographs were obtained on a Nikon A1RSi confocal microscope using either a Plan Apo 60X 1.4 NA or a Plan Apo 100X 1.49 NA oil immersion objective using the Nikon Elements 4.0 software package. Micrographs were processed using Nikon Elements or ImageJ (NIH). CID and FISH spot counts were performed on maximum z-projections from z-stack images using the counting software in Nikon Elements. 3D FISH distance measurements were performed manually in Nikon Elements using the 3D distance measuring tool by scanning through each Z-slice. The centroid of each FISH signal would be marked and the shortest 3D pairwise distance would then be calculated. For scenarios where the chromosomes are unpaired, resulting in two foci per probe (6 FISH signal total), pairwise distance measurements were performed on the signals that were closer to each other. The assumption made is that in the situation where the chromosomes are unpaired, it is more likely that the three signals closer to one another are from the same chromosome and that the further three signals are from the other chromosome, as previously described for similar measurements made in mammalian and *C*. *elegans* cells [26,58,59].

Salivary gland polytene squash intensity analysis was performed by obtaining multiple fields of chromosomes from different genotypes on the same slide. Experimental genotypes (CK1α mutants and RNAi) were squashed on the same slide as their respective controls (*Oregon R* for mutants and *43B>Gal4* for RNAi) such that they could be imaged using identical settings for downstream analyses [43]. Multiple fields (5–6) of chromosomes were imaged for each genotype in both DAPI and Cap-H2 channels. Images used for fluorescence intensity measurements were captured such that pixel saturation was limited for all channels. Images were then analyzed using ImageJ as follows: First, all image sets were split into separate channels (DAPI and Cap-H2) and converted to gray scale. Secondly, the mean/average intensity value for the entire DAPI image was measured. Background fluorescence was subtracted by measuring a “blank” square from the same DAPI image and subtracting mean background intensity of this square from the mean grey value of the entire image (DAPI_mean intensity_—Background_mean intensity_ = DAPI_corrected_). This produced a background subtracted intensity value for the DAPI image (DAPI_corrected_). Next, the same process was performed for the image using the Cap-H2 image in grey scale (Cap-H2_correct_). Then, the ratio of Cap-H2 to DAPI was calculated (Cap-H2_corrected_ ÷ DAPI_corrected_ = Chromatin Cap-H2) for each set of images. These values were then averaged for all the image fields obtained for each genotype. Changes in chromatin bound Cap-H2 levels were calculated by comparing the experimental genotype average ratio to its respective control average ratio (Chromatin Cap-H2_CK1α Mutants_ ÷ Chromatin Cap-H2_Controls_ = Change in chromatin bound Cap-H2). Values in Fig. 6D represent fold-changes in Cap-H2 as compared to their respective controls. Statistical analyses were performed in Microscoft Excel using a two-tailed student t-test assuming unequal variance.

Mitotic indexes/percentage of cells staining positive for phosphorylated-histone-H3 were assessed by using CellProfiler Cell Image Analysis Software (http://www.cellprofiler.org; Broad Insitute). Micrographs were captured on a Nikon Eclipse E800 Epifluorescent upright microscope using a Nikon Plan Fluor 20X 0.5 NA objective with Olympus DP controller software.

### Immunoblotting

For Fig. 7A-B, cell extracts were obtained by pelleting and lysing cells in PBT with protease inhibitor (Roche). Protein concentration was calculated by performing the Bradford protein assay (Bio-Rad Laboratories). Laemmli sample buffer was then added to extracts and boiled for 5 minutes prior to loading onto denaturing gel. For Fig. 7, antibodies used are as follows: mouse anti-GFP (JL8 Takara Bio Inc.), guinea pig anti-Slimb (Brownlee et al, 2011), mouse anti-armadillo (N2 7A1; Drosophila Hybridoma Bank), mouse anti-alpha-tubulin (Dm1; Sigma-Aldrich), mouse anti-lamin Dm0 (ADL84.12; Drosophila Hybridoma Bank), and rabbit anti-Hisone-H3 (06–755; EMD Millipore).

### Immunoprecipitations

Immunoprecipitations were performed as previously described [20]. GFP-binding protein (GBP; [60]) was fused to the Fc domain of human IgG (pIg-Tail; R&D Systems), tagged with His6 in pET28a (EMD Millipore), expressed in *E*. *coli*, and purified on Talon resin (Takara Bio Inc.) according to manufacturer’s instructions. GBP was bound to Protein A–coupled Sepharose, cross-linked to the resin using dimethyl pimelimidate, and rocked for 1 h at 22°C; the coupling reaction was then quenched in 0.2 M ethanolamine, pH 8.0, and rocked for 2 h at 22°C. Antibody or GBP-coated beads were washed three times with 1.5 ml of cell lysis buffer (CLB; 100 mM Tris, pH 7.2, 125 mM NaCl, 1 mM DTT, 0.1% Triton X-100, and 1X Protease Inhibitor (Roche). S2, Kc, and Kc stable line expressing inducible Cap-H2-EGFP cells were treated with RNAi for 6 days, as described above in “Cell culture and double-stranded RNAi.” On day 4, cells were transfected with inducible GFP tag only, Cap-H2-EGFP, and/or 3x-FLAG-Ubiquitin. On day 5, transfected cells were induced with 1 mM CuSO4. After 24 h, transfected cells were lysed in CLB, clarified by centrifugation, and then diluted to 2–5 mg/ml in CLB. Antibody-coated beads were mixed with lysate in 1mL total volume for 90 min at 4°C, washed three times with CLB, and then boiled in Laemmli sample buffer.

### Cellular fractionations

Kc cells stably expressing an inducible Cap-H2-EGFP (provided by the Wu lab) were treated with RNAi as described above but in T75 flasks. Cells were induced 24h prior to harvesting. On day 6, cells were harvested and then fractionated into whole cell lysate (WCL), cytoplasmic (S2), nuclear-soluble (S3), and chromatin (P3) fractions as described previously [20,61]. In brief, ~10^7^ cells were collected, washed with cold PBS, and resuspended at 4 × 10^7^ cells/ml in buffer A: 10 mM Hepes, pH 7.9, 10 mM KCl, 1.5 mM MgCl2, 0.34 M sucrose, 10% glycerol, 1 mM DTT, and protease inhibitor cocktail (Roche). Cells were lysed by the addition of 0.1% Triton X-100 and then incubated on ice for 8 min (fraction WCL). Nuclei were collected by centrifugation (5 min, 1,300 *g*, 4°C), the initial supernatant was further cleared by high-speed centrifugation (5 min, 20,000 *g*, 4°C), and the final supernatant was collected (fraction S2). The pelleted nuclei were washed once in buffer A, and nuclear membranes were lysed for 30 min in 3 mM EDTA, 0.2 mM EGTA, 1 mM DTT, and protease inhibitor cocktail (Roche) (buffer B). The insoluble chromatin (fraction P3) and soluble (fraction S3) fractions were separated by centrifugation (5 min, 1,700 *g*, 4°C). The insoluble chromatin pellet was washed once with buffer B and resuspended in SDS loading buffer. The protein concentration of each fraction was determined by a Bradford’s assay (Bio-Rad Laboratories), and 20 μg of protein from each fraction was immunoblotted.

### Flow cytometry

Flow cytometry and DNA content analysis on RNAi treated S2 cells was performed as previously described[62]. RNAi treated S2 cells (10^6^) were pelleted at 1,000 *g* for 5 min, resuspended in 0.5 ml PBS, and vortexed while intermittently adding 0.5 ml of cold 100% ethanol. Fixed cells were incubated on ice for 20 min, pelleted (1,000 *g* for 5 min), and resuspended in a 0.5 ml propidium iodide (PI)-RNase solution (50 μg/ml PI + 100 μg/ml RNase Type1 I-A [QIAGEN] in PBS). After 20 min, cells were passed through a 12 × 75–mm flow cytometry tube (Falcon; Thermo Fisher Scientific). Cytometric analysis was performed in the Arizona Cancer Center/Arizona Research Laboratories Division of Biotechnology Cytometry Core Facility using a FACScan flow cytometer (BD) equipped with an air-cooled 15-mW argon ion laser tuned to 488 nm. List mode data files consisting of 10,000 cells gated on forward scatter versus side scatter were acquired and analyzed using CellQuest Pro software (BD).

### *Drosophila* strains

Fly crosses were performed on yeast/molasses/cornmeal media and kept at 25°. *CK1α^G0492^* (Bloomington Stock Center # 12303), *CK1α^EP1555^* (Bloomington Stock Center # 17009), *CK1α^8B12^* (gift from Yashi Ahmed)[40], *CK1α^RNAi^* *VALIUM10* (Harvard TRiP line: JF7192, Bloomington Stock Center # 25786), *SMC4^k00819^* (Bloomington Stock Center # 10831) [63], *Cap-H2^Z3–0019^* [16], *43B>Gal4* salivary gland specific driver (gift from Patrick O'Farrel)[44], *Slimb^3A1^*, *Slimb^UU11^* alleles were previously described[20].

## Supporting Information

S1 Fig CK1α depletion in Kc cells does not affect cell viability. (A) Micrographs of RNAi treated Kc cells stained with propidium iodide (green) to mark dead cells and counterstained with DAPI (DNA, red). Dead cells are marked by presence of both DAPI and propidium iodide staining (yellow). (B) Histogram showing percentage of dead cells marked by propidium iodide staining in Kc cells after RNAi depletion of indicated protein. CK1α depletion does not significantly affect cell viability, while SMC2 depletion significantly increases percentage of dead cells after 4 days of RNAi treatment. n = 600–700 cells per treatment. * = p-value < 0.0005 (calculated by using students’ t-test in MS Excel). Error bars indicate SEM. (A) Images are from single z-slices.
(TIF)

S2 Fig CK1α RNAi depletion causes abnormal dispersal of centromeric protein CID, chromosome compaction, and chromosome unpairing in *Drosophila* cultured cells. (A) Micrographs of RNAi treated S2 cells immunostained for centromeric protein (CID) and counterstained for DNA (DAPI, blue). CK1α depletion induces abnormal centromere dispersal, which is suppressed by double RNAi of CK1α + Cap-H2. (B) Histogram showing average number of CID spots per S2 nucleus after RNAi depletion of the indicated protein (n = 100–142 cells per treatment). CK1α depletion results in a significant increase in number of CID spots, which is suppressed with codepletion of Cap-H2. Statistical comparisons are between RNAi treatments and control, unless denoted by horizontal line between bars. (C) Histogram showing average number of CID spots per nucleus after RNAi depletion of the indicated protein in Kc cells. Suppression of increase in CID spots in CK1α-RNAi is suppressed by CK1α + Cap-H2 RNAi but not CK1α + Barren RNAi (n = 115–180 cells per treatment). Statistical comparisons are between RNAi treatments and control, unless denoted by horizontal line between bars. (D) Micrographs of RNAi treated Kc cells stained with FISH probes specific to two locations on the X Chromosome: X1 (green) and X2 (Red) and counterstained for DNA (DAPI, blue). CK1α RNAi results in increased chromosome compaction and unpairing of chromosomes (quantification in Fig. 3G,H). (E) Histogram (modified from Fig. 3G) showing the average number of FISH spots per nucleus in RNAi depleted Kc cells (n = 50–110 cells per treatment). CK1α +Barren RNAi does not significantly suppress the increase in the number of FISH spots seen in CK1α RNAi. Statistical comparisons are between RNAi treatments and CK1α RNAi. (F) Micrographs of RNAi treated Kc cells stained with FISH probes specific to heterochromatic regions on Chromosome 2R (green), 3R (red), and counterstained for DNA (DAPI, blue). CK1α RNAi results in unpairing of heterochromatic loci (quantification in Fig. 3M). N.S. = No significance. * = p-value < 8.5x10^−3^ (calculated by using students’ t-test in MS Excel). Error bars indicate SEM. (A,D,F) Maximum projection image of multiple z-slices. Scale bar, 5μm.
(TIF)

S3 Fig CK1α depletion in Kc cells does not increase mitotic index or cell ploidy. (A) Micrographs of RNAi treated Kc cells immunostained for Phosphorylated Histone H3 (green), a mitotic marker, and counterstained for DNA (DAPI, magenta). CK1α depletion reduces the number of cells undergoing mitosis. (B) Histogram showing average mitotic indexes of Kc cells after RNAi treatments. CK1α depletion significantly reduces the amount of cells undergoing mitosis. This reduction is suppressed by co-depletion of CK1α and Cap-H2; (n = 3900–7100 cells per treatment). p-value = * = 0.046, ** = 0.0014, *** = 7.9x10^−6^ (calculated by using students’ t-test in MS excel). Statistical comparisons are between RNAi treatments and control, unless denoted by horizontal line between bars. Error bars indicate SEM. (C) Histograms of DNA fluorescence intensity (x axis) and cell number (y axis) from flow cytometry on RNAi treated S2 cells. Increased proportion of cells in G1-phase in CK1α depleted cells. (A) Images are from single z-slice. Scale bar, 50μm.
(TIF)

S4 Fig CK1α and Slimb double heterozygous mutants do not increase unpairing of salivary gland nuclei. (A) Micrographs of salivary gland nuclei from control wild-type larvae (*Oregon-R*), Slimb heteroyzygotes (*Slimb^UU11^*/+ and *Slimb^3A1^*/+), CK1α heterozygote (*CK1α^8B12^*/+), and Slimb/CK1α double heterozygotes (*CK1α^8B12^*/+; *Slimb^UU11^*/+ and *CK1α^8B12^*/+; *Slimb^3A1^*/+) were stained with a FISH probe specific to a region of Chromosome 2L (green) and counterstained with DAPI (DNA, blue). Chromosomes are highly paired in control nuclei with single and double heterozygous mutations in CK1α and/or Slimb having non-significant effects on chromosome pairing status. (B) Histogram showing average number of FISH spots per nucleus in salivary glands from (A); (n = 26–41 nuclei per genotype). Statistics calculated by using students’ t-test in excel. Error bars indicate SEM. (A) Maximum projection image of multiple z-slices. Scale, 10μm.
(TIF)

S5 Fig Condensin mutant nurse cell polytene defect is suppressed by *CK1α^G0492^* */+* mutation in vivo. (A-D) Micrographs of stage 10 nurse cells from control (triple balancer) (A), *CK1α^G0492^*/+ heterozygous mutant (B), condensin II loss of function mutant (*SMC4^k00819^*/+; *Cap-H2^z3–0019^*/+) (C), and *CK1α^G0492^*/+ heterozygote in condensin II loss of function background (*CK1α^G0492^* */+; SMC4^k00819^*/+; *Cap-H2^z3–0019^*/+) (D) were stained with a FISH probe specific to Chromosome 2L (green) and counterstained with DAPI (DNA, blue). (E) Histogram showing the average number of FISH spots for each probe in stage 10 nurse cells. n = 23–31 nurse cells per genotype. Error bars indicate SEM. p-value = * < 0.0001 (calculated by using students’ t-test in excel). (A-D) Maximum projection image of multiple z-slices. Scale, 20μm.
(TIF)

S6 Fig CK1α, PKA, and GSK3β depletion does not affect Slimb and Cap-H2 interaction in S2 cells. Immunoprecipitations and immunoblots from RNAi treated S2 cells, transiently transfected with inducible EGFP as a negative control or inducible Cap-H2-EGFP. Anti-Cap-H2-EGFP immunoprecipitates Slimb in both control and CK1α depleted cells expressing Cap-H2-EGFP. GFP tag only transfected cells did not immunoprecipitate Slimb. Anti-armadillo was used to verify CK1α depletion and anti-Lamin-Dm0 was used as protein loading control.
(TIF)

S1 Table Primer sequences to generate dsRNAs used in the study. All primers begin with the T7 promoter sequence: 5'-TAATACGACTCACTATAGGG-3', followed by the gene specific primer sequence. Control dsRNA was generated using a plasmid encoding pEGFP-N1 as template. NA = Not applicable.
(TIF)
