# Supplementary figures and images for: Drosophila Casein Kinase I Alpha Regulates Homolog Pairing and Genome Organization by Modulating Condensin II Subunit Cap-H2 Levels
Source: PLoS Genet. 2015 Feb 27;11(2):e1005014. doi: 10.1371/journal.pgen.1005014 (PMC4344196; doi:10.1371/journal.pgen.1005014)

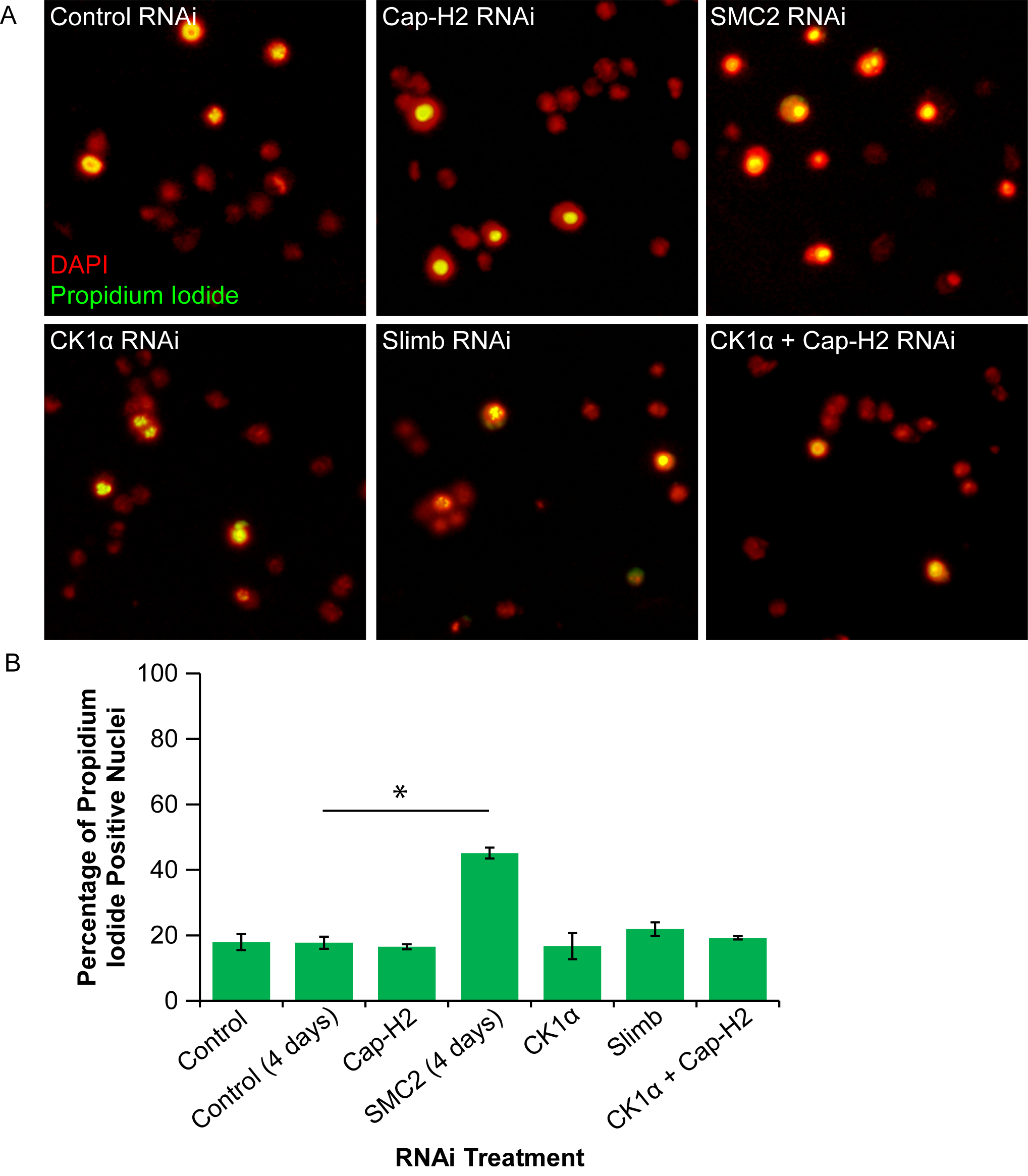

Supplement: S1 Fig — (A) Micrographs of RNAi treated Kc cells stained with propidium iodide (green) to mark dead cells and counterstained with DAPI (DNA, red). Dead cells are marked by presence of both DAPI and propidium iodide staining (yellow). (B) Histogram showing percentage of dead cells marked by propidium iodide staining in Kc cells after RNAi depletion of indicated protein. CK1α depletion does not significantly affect cell viability, while SMC2 depletion significantly increases percentage of dead cells after 4 days of RNAi treatment. n = 600–700 cells per treatment. * = p-value < 0.0005 (calculated by using students’ t-test in MS Excel). Error bars indicate SEM. (A) Images are from single z-slices. (TIF) [file pgen.1005014.s001.tif]

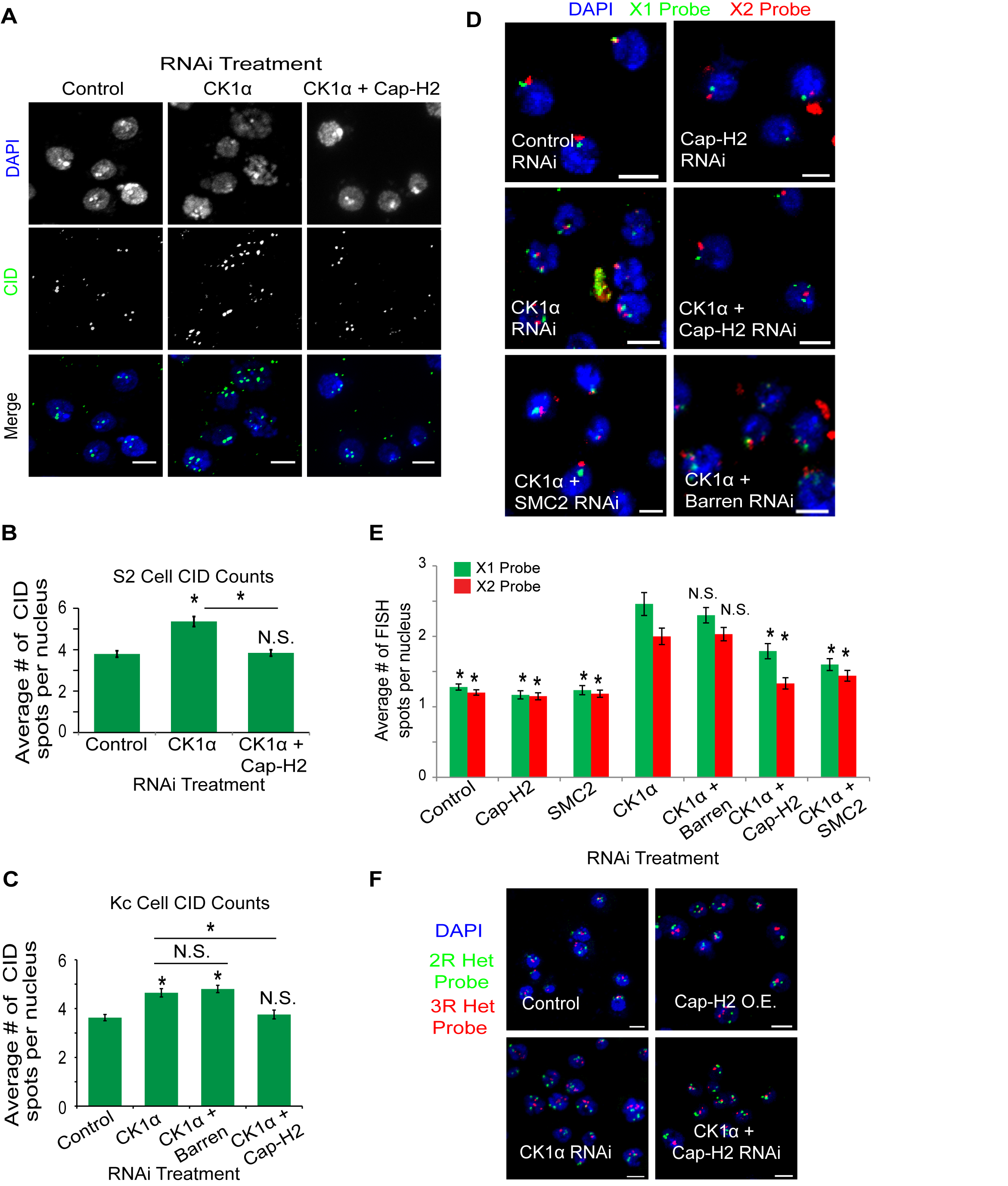

Supplement: S2 Fig — (A) Micrographs of RNAi treated S2 cells immunostained for centromeric protein (CID) and counterstained for DNA (DAPI, blue). CK1α depletion induces abnormal centromere dispersal, which is suppressed by double RNAi of CK1α + Cap-H2. (B) Histogram showing average number of CID spots per S2 nucleus after RNAi depletion of the indicated protein (n = 100–142 cells per treatment). CK1α depletion results in a significant increase in number of CID spots, which is suppressed with codepletion of Cap-H2. Statistical comparisons are between RNAi treatments and control, unless denoted by horizontal line between bars. (C) Histogram showing average number of CID spots per nucleus after RNAi depletion of the indicated protein in Kc cells. Suppression of increase in CID spots in CK1α-RNAi is suppressed by CK1α + Cap-H2 RNAi but not CK1α + Barren RNAi (n = 115–180 cells per treatment). Statistical comparisons are between RNAi treatments and control, unless denoted by horizontal line between bars. (D) Micrographs of RNAi treated Kc cells stained with FISH probes specific to two locations on the X Chromosome: X1 (green) and X2 (Red) and counterstained for DNA (DAPI, blue). CK1α RNAi results in increased chromosome compaction and unpairing of chromosomes (quantification in Fig. 3G,H). (E) Histogram (modified from Fig. 3G) showing the average number of FISH spots per nucleus in RNAi depleted Kc cells (n = 50–110 cells per treatment). CK1α +Barren RNAi does not significantly suppress the increase in the number of FISH spots seen in CK1α RNAi. Statistical comparisons are between RNAi treatments and CK1α RNAi. (F) Micrographs of RNAi treated Kc cells stained with FISH probes specific to heterochromatic regions on Chromosome 2R (green), 3R (red), and counterstained for DNA (DAPI, blue). CK1α RNAi results in unpairing of heterochromatic loci (quantification in Fig. 3M). N.S. = No significance. * = p-value < 8.5x10^−3^ (calculated by using students’ t-test in MS Excel). Error bars indicate SEM. [file pgen.1005014.s002.tif]

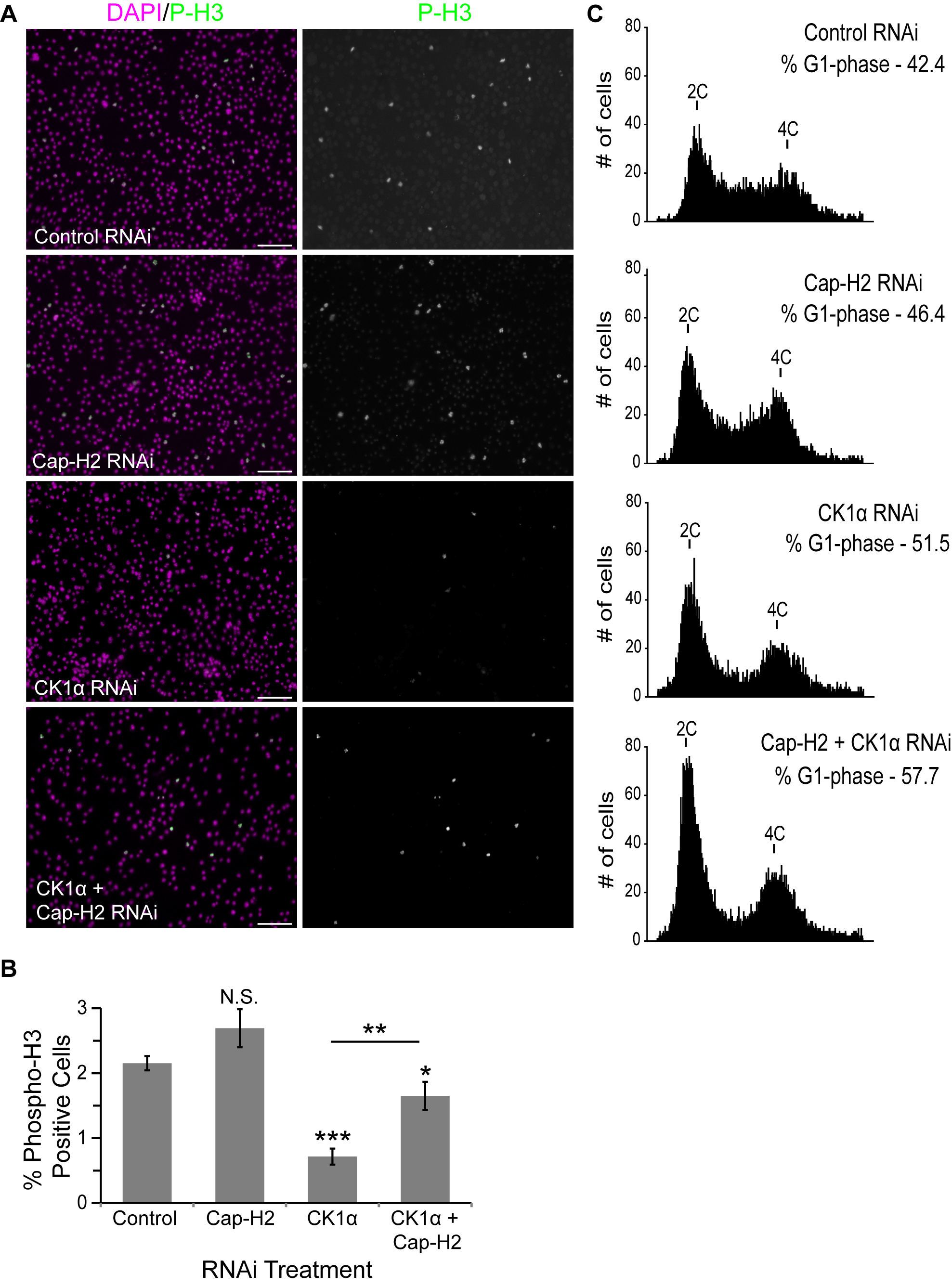

Supplement: S3 Fig — (A) Micrographs of RNAi treated Kc cells immunostained for Phosphorylated Histone H3 (green), a mitotic marker, and counterstained for DNA (DAPI, magenta). CK1α depletion reduces the number of cells undergoing mitosis. (B) Histogram showing average mitotic indexes of Kc cells after RNAi treatments. CK1α depletion significantly reduces the amount of cells undergoing mitosis. This reduction is suppressed by co-depletion of CK1α and Cap-H2; (n = 3900–7100 cells per treatment). p-value = * = 0.046, ** = 0.0014, *** = 7.9x10^−6^ (calculated by using students’ t-test in MS excel). Statistical comparisons are between RNAi treatments and control, unless denoted by horizontal line between bars. Error bars indicate SEM. (C) Histograms of DNA fluorescence intensity (x axis) and cell number (y axis) from flow cytometry on RNAi treated S2 cells. Increased proportion of cells in G1-phase in CK1α depleted cells. (A) Images are from single z-slice. Scale bar, 50μm. (TIF) [file pgen.1005014.s003.tif]

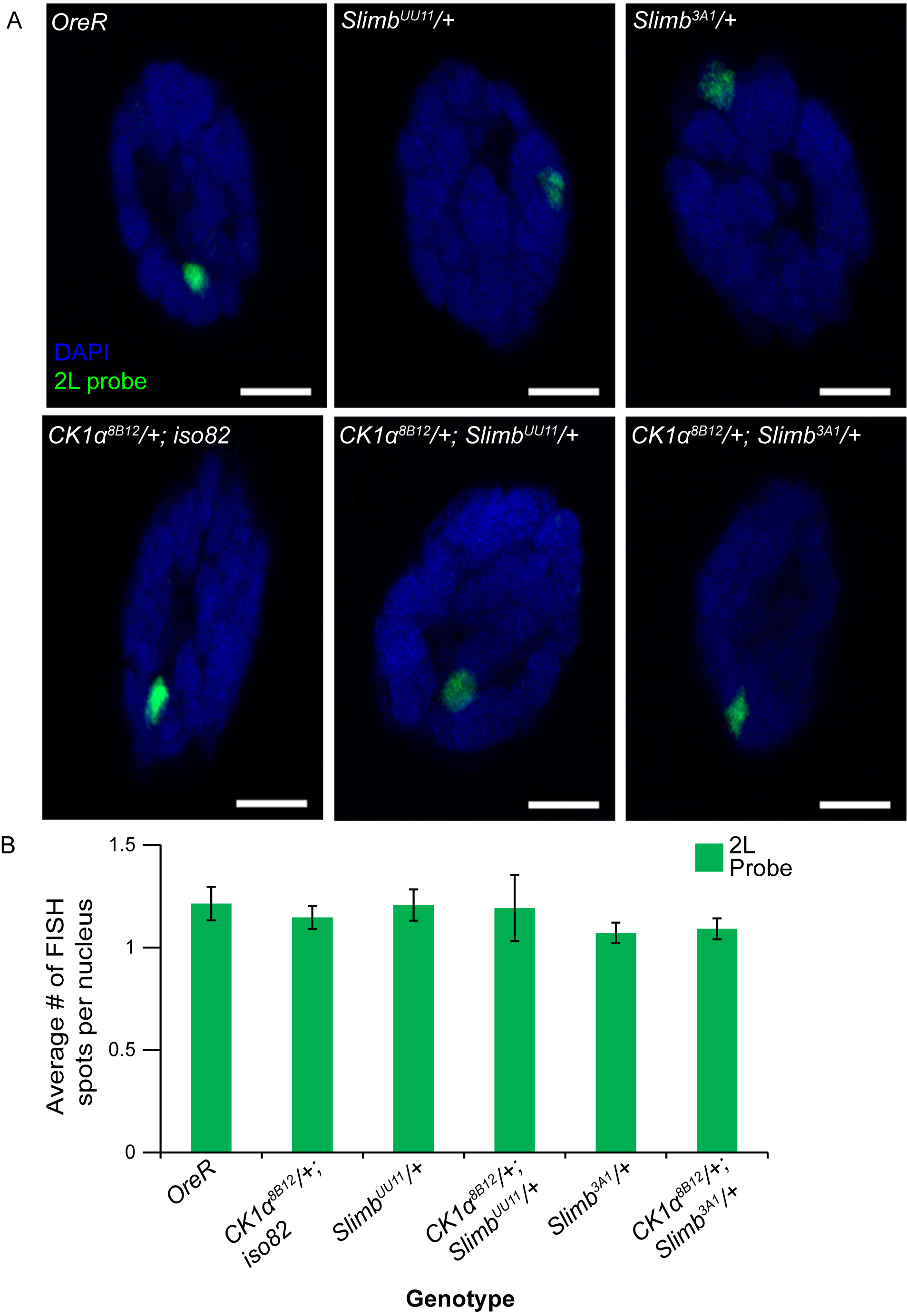

Supplement: S4 Fig — (A) Micrographs of salivary gland nuclei from control wild-type larvae (Oregon-R), Slimb heteroyzygotes (SlimbUU11/+ and Slimb3A1/+), CK1α heterozygote (CK1α8B12/+), and Slimb/CK1α double heterozygotes (CK1α8B12/+; SlimbUU11/+ and CK1α8B12/+; Slimb3A1/+) were stained with a FISH probe specific to a region of Chromosome 2L (green) and counterstained with DAPI (DNA, blue). Chromosomes are highly paired in control nuclei with single and double heterozygous mutations in CK1α and/or Slimb having non-significant effects on chromosome pairing status. (B) Histogram showing average number of FISH spots per nucleus in salivary glands from (A); (n = 26–41 nuclei per genotype). Statistics calculated by using students’ t-test in excel. Error bars indicate SEM. (A) Maximum projection image of multiple z-slices. Scale, 10μm. (TIF) [file pgen.1005014.s004.tif]

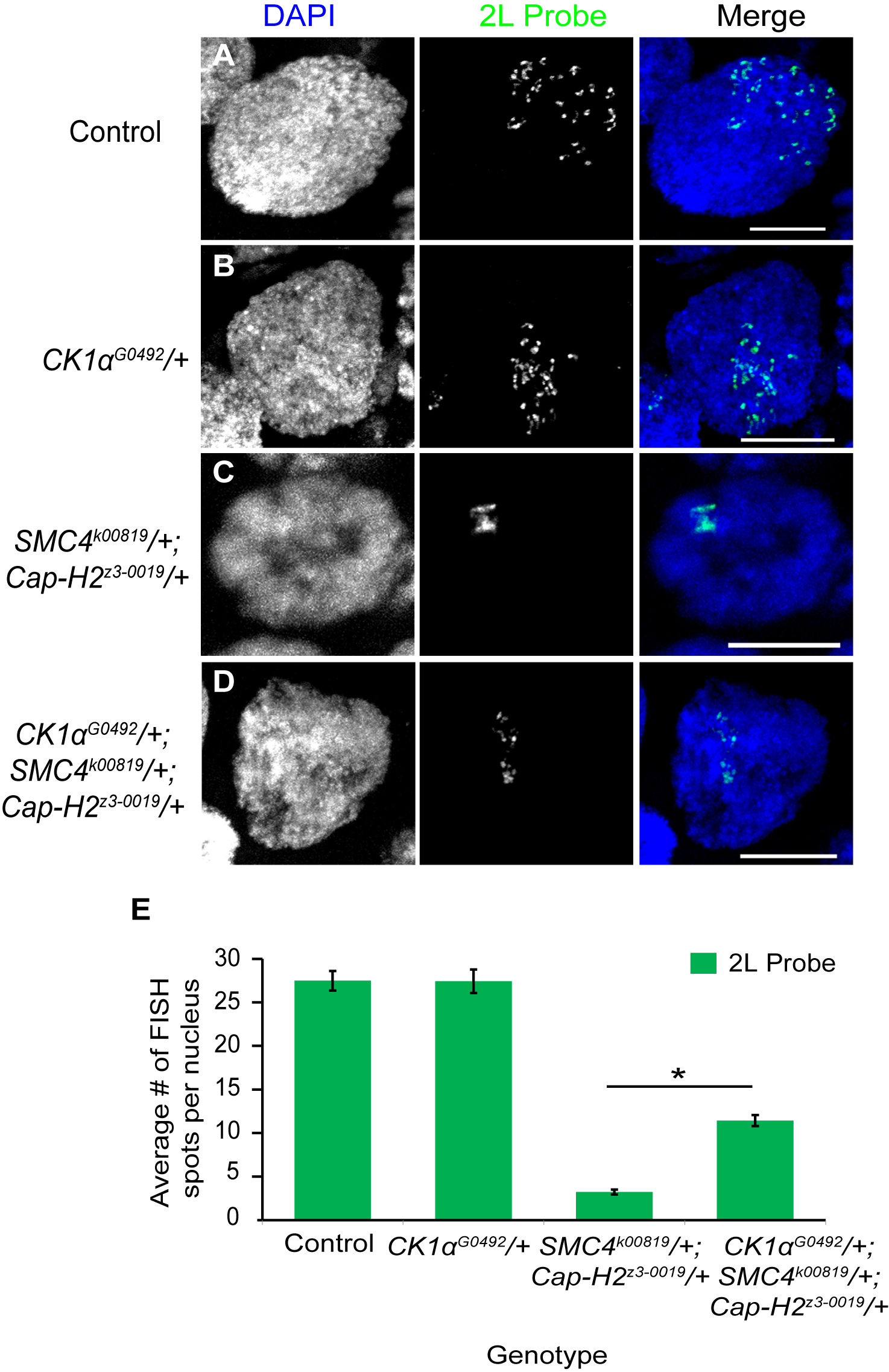

Supplement: S5 Fig — (A-D) Micrographs of stage 10 nurse cells from control (triple balancer) (A), CK1αG0492/+ heterozygous mutant (B), condensin II loss of function mutant (SMC4k00819/+; Cap-H2z3–0019/+) (C), and CK1αG0492/+ heterozygote in condensin II loss of function background (CK1αG0492 /+; SMC4k00819/+; Cap-H2z3–0019/+) (D) were stained with a FISH probe specific to Chromosome 2L (green) and counterstained with DAPI (DNA, blue). (E) Histogram showing the average number of FISH spots for each probe in stage 10 nurse cells. n = 23–31 nurse cells per genotype. Error bars indicate SEM. p-value = * < 0.0001 (calculated by using students’ t-test in excel). (A-D) Maximum projection image of multiple z-slices. Scale, 20μm. (TIF) [file pgen.1005014.s005.tif]

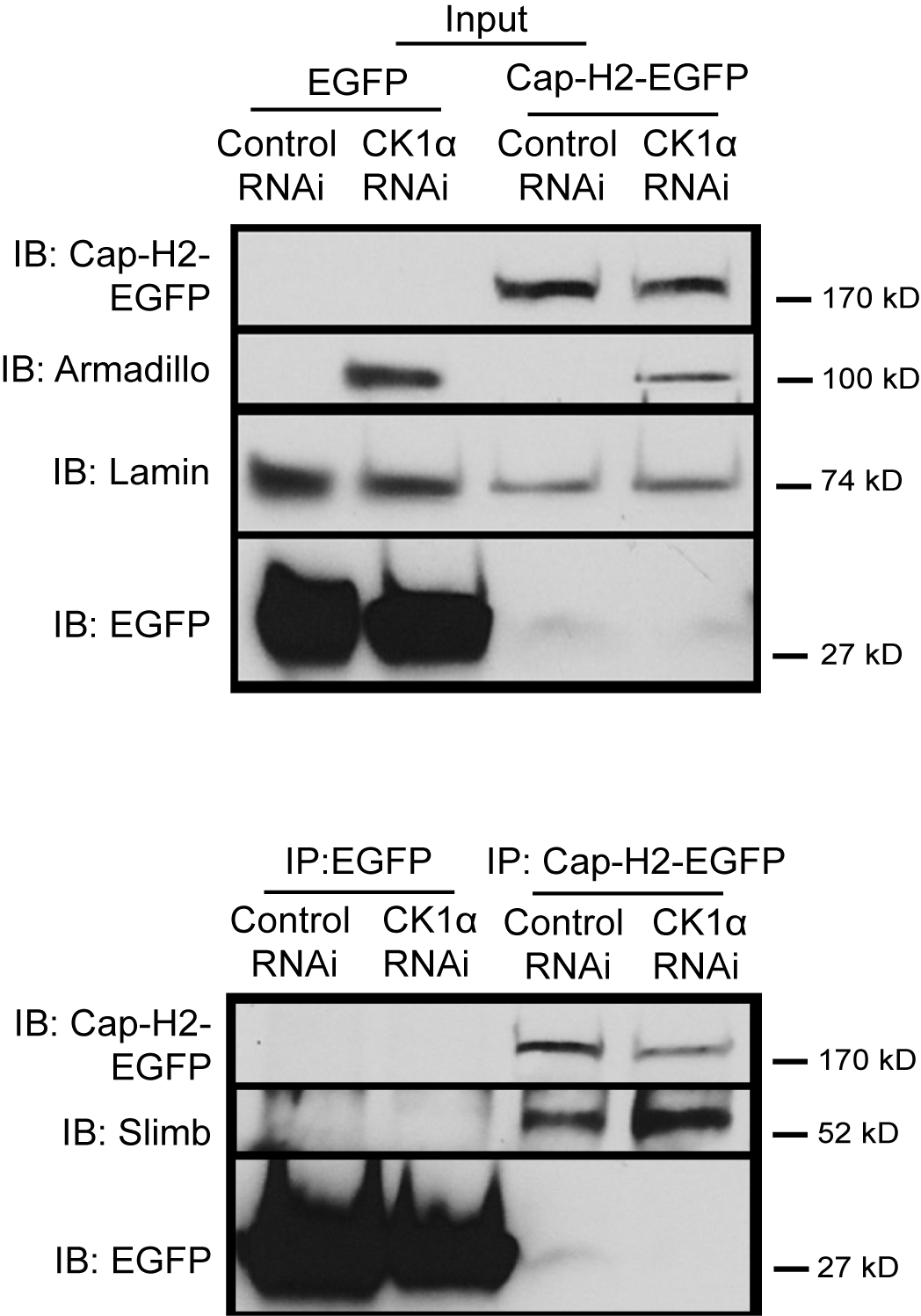

Supplement: S6 Fig — Immunoprecipitations and immunoblots from RNAi treated S2 cells, transiently transfected with inducible EGFP as a negative control or inducible Cap-H2-EGFP. Anti-Cap-H2-EGFP immunoprecipitates Slimb in both control and CK1α depleted cells expressing Cap-H2-EGFP. GFP tag only transfected cells did not immunoprecipitate Slimb. Anti-armadillo was used to verify CK1α depletion and anti-Lamin-Dm0 was used as protein loading control. (TIF) [file pgen.1005014.s006.tif]

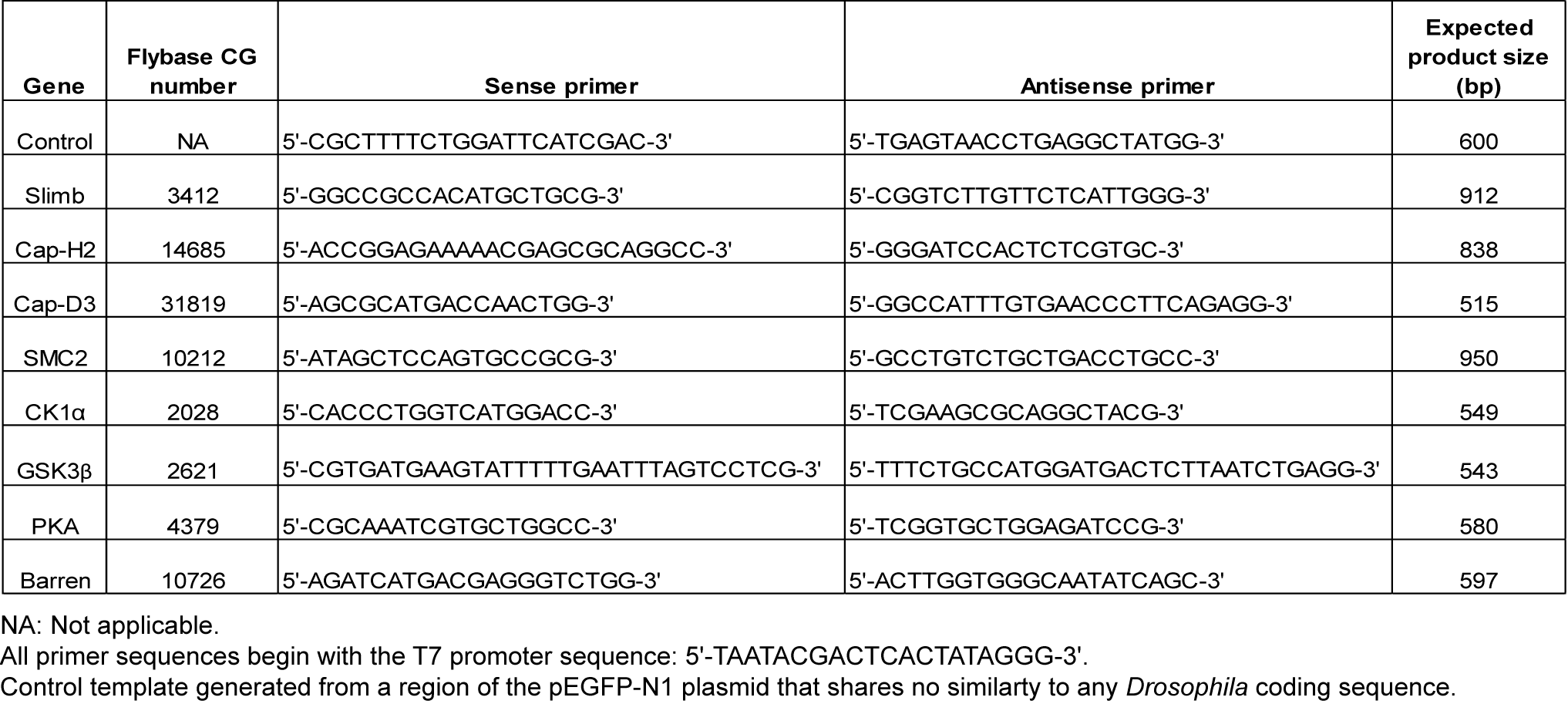

Supplement: S1 Table — All primers begin with the T7 promoter sequence: 5'-TAATACGACTCACTATAGGG-3', followed by the gene specific primer sequence. Control dsRNA was generated using a plasmid encoding pEGFP-N1 as template. NA = Not applicable. (TIF) [file pgen.1005014.s007.tif]
